# Supplementary material for: Sick leave or work sick? Examining the antecedents and conceptualizations of presenteeism and absenteeism among teleworkers during COVID-19: A scoping review
Source: PLOS Ment Health. 2025 May 13;2(5):e0000300. doi: 10.1371/journal.pmen.0000300 (PMC12798164; doi:10.1371/journal.pmen.0000300)
Supplement: S1 Data — (DOCX) [file pmen.0000300.s001.docx]

| Databases | Search Terms |
| --- | --- |
| MEDLINE | Presenteeism AND Definition; Presenteeism AND Telework; Absenteeism AND Definition; Absenteeism AND Telework; Sickness Presenteeism AND Definition; Sickness Presenteeism AND Telework; Sickness Absenteeism AND Definition; Sickness Absenteeism AND Telework |
| CINAHL | Presenteeism AND Definition; Presenteeism AND Telework; Absenteeism AND Definition; Absenteeism AND Telework; Sickness Presenteeism AND Definition; Sickness Presenteeism AND Telework; Sickness Absenteeism AND Definition; Sickness Absenteeism AND Telework |
| PsycINFO | Presenteeism AND Definition; Presenteeism AND Telework; Absenteeism AND Definition; Absenteeism AND Telework; Sickness Presenteeism AND Definition; Sickness Presenteeism AND Telework; Sickness Absenteeism AND Definition; Sickness Absenteeism AND Telework |
| ABI Inform Global | Presenteeism AND COVID-19; Presenteeism AND Remote Working; Absenteeism AND COVID-19; Absenteeism AND Remote Working; Sickness Presenteeism AND COVID-19; Sickness Presenteeism and Remote Working; Sickness Absenteeism AND COVID-19; Sickness Absenteeism AND Remote Working |
| Scopus | Presenteeism AND COVID-19; Presenteeism AND Remote Working; Absenteeism AND COVID-19; Absenteeism AND Remote Working; Sickness Presenteeism AND COVID-19; Sickness Presenteeism and Remote Working; Sickness Absenteeism AND COVID-19; Sickness Absenteeism AND Remote Working |
| Web of Science and Business Source Premier | Presenteeism AND Definition; Presenteeism AND Telework; Absenteeism AND Definition; Absenteeism AND Telework; Sickness Presenteeism AND Definition; Sickness Presenteeism AND Telework; Sickness Absenteeism AND Definition; Sickness Absenteeism AND Telework |
