# Supplementary material for: Sick leave or work sick? Examining the antecedents and conceptualizations of presenteeism and absenteeism among teleworkers during COVID-19: A scoping review
Source: PLOS Ment Health. 2025 May 13;2(5):e0000300. doi: 10.1371/journal.pmen.0000300 (PMC12798164; doi:10.1371/journal.pmen.0000300)
Supplement: Attachment — Submitted filename: Response to Reviewers.docx [file pmen.0000300.s004.docx]

Comments to the Author

1. Does this manuscript meet PLOS Mental Health’s publication criteria? Is the manuscript technically sounds, and does the data support the conclusions? The manuscript must describe methodologically and ethically rigorous research with conclusions that are appropriately drawn based on the data presented

Reviewer 1: Yes

Reviewer 2: Partly

2. Has the statistical analysis been performed appropriately and rigorously?

Reviewer 1: I don't know

Reviewer 2: No

3. Have the authors made all data underlying the findings in their manuscript fully available (please refer to the Data Availability Statement at the start of the manuscript PDF file)?

   The [PLOS Data policy](https://can01.safelinks.protection.outlook.com/?url=https%3A%2F%2Fc05y1x9s.r.us-east-2.awstrack.me%2FL0%2Fhttps%3A%252F%252Fjournals.plos.org%252Fmentalhealth%252Fs%252Fdata-availability%2F1%2F010f019177c2c76b-ee7d87b0-0aae-477c-8345-34823a693ff7-000000%2Fi9p0bu0rhWWEeMlphSmIyC8CKgY%3D173&data=05%7C02%7Cmaryna.mazur%40mail.utoronto.ca%7C6761860dc8e44f28683908dcc2a3667d%7C78aac2262f034b4d9037b46d56c55210%7C0%7C0%7C638599255619770348%7CUnknown%7CTWFpbGZsb3d8eyJWIjoiMC4wLjAwMDAiLCJQIjoiV2luMzIiLCJBTiI6Ik1haWwiLCJXVCI6Mn0%3D%7C0%7C%7C%7C&sdata=b8pDYBPKvJ9C8m5nyD%2BpNH9HXmAELQS8CSUDfJEMn3Y%3D&reserved=0) requires authors to make all data underlying the findings described in their manuscript fully available without restriction, with rare exception. The data should be provided as part of the manuscript or its supporting information, or deposited to a public repository. For example, in addition to summary statistics, the data points behind means, medians and variance measures should be available. If there are restrictions on publicly sharing data—e.g. participant privacy or use of data from a third party—those must be specified.

Reviewer 1: No

Reviewer 2: Yes

4. Is the manuscript presented in an intelligible fashion and written in standard English?

   PLOS Mental Health does not copyedit accepted manuscripts, so the language in submitted articles must be clear, correct, and unambiguous. Any typographical or grammatical errors should be corrected at revision, so please note any specific errors here.

Reviewer 1: Yes

Reviewer 2: No

**Reviewer 1:**

**Comment 1:** The purpose of this paper's research is to define presenteeism and absenteeism in the (tele)workplace and to systematically identify the factors contributing to their occurrence.

**Response to Comment 1:** That is correct. Thank you for the comment.

**Comment 2:** The method is appropriate as it extracts definitions of presenteeism and absenteeism in the (tele)workplace during COVID-19 from seven online databases.

**Response to Comment 2:** Thank you for the comment.

**Comment 3:** On the other hand, the researchers consulted experts to classify the antecedent factors of presenteeism and absenteeism, but it is difficult to understand how they were classified, so I would like to see them summarized in a table.

**Response to Comment 3:** Thank you for the suggestion. We have added a table to our manuscript highlighting the themes and sub-themes for factors associated with presenteeism and absenteeism.

**Comment 4:** I don't think it's new to conclude that antecedent factors at the organizational, environmental, and individual levels interact with each other. In the next study, it is hoped that statistical methods will be used to further analyze the content of individual factors.

**Response to Comment 4:** Thank you for highlighting this. We included in our future directions section that this could be a potential research avenue.

**Comment 5:** It is stated that there were few papers that reported the definition of absenteeism, but this is not surprising since COVID-19 has led to an increase in people working from home and presenteeism, where people work online instead of being absent from work. It would be necessary to mention that.

**Response to Comment 5:** Thank you for the comment. your recommendation of *“It is stated that there were few papers that reported the definition of absenteeism, but this is not surprising since COVID-19 has led to an increase in people working from home and presenteeism, where people work online instead of being absent from work”* has now been incorporated in the manuscript in 4.4.1 limitations section. Your recommendation reinforces the lack of studies exploring absenteeism.

**Comment 6:** Reading the entire paper, absenteeism was a problem before the COVID-19 outbreak, but during the COVID-19 outbreak, telework is required and presenteeism has increased, and presenteeism is undesirable from the standpoint of worker health and work efficiency. I thought this would be a consideration.

**Response to Comment 6:** Thank you for bringing this into light, we clarified this in our manuscript.

**Comment 7:** This paper shows that telework due to the COVID-19 pandemic has resulted in various problems as people have been forced to work from home without a proper work environment. Since telework may be suitable for some workers to achieve work-life balance, it would be desirable to see future research on how employers can appropriately manage telework.

**Response to Comment 7:** Thank you for the comment. We have incorporated your suggestion in the future directions section of the discussion, section 4.4.3.

**Reviewer 2:**

**Comment 1:** Given the specialist focus of the journal, the authors should more overtly link their core focus (antecedents of presenteeism and absenteeism amongst teleworkers) to mental health. This could be achieved through insertion of a sentence or two in the opening section, with relevant citations, as well as in the discussion and conclusion sections.

**Response to Comment 1:**

**Comment 2:** Since the Social Determinants of Health model is ultimately used to frame the findings of the review, this model should also be introduced briefly in the opening section of the manuscript or alternatively referred to in the methods section.

**Response to Comment 2:** We have discussed the framework near the end of the abstract by stating “We found that each of these antecedents relate to one another through the social determinants of health framework.” We have now included an introduction to the framework in the manuscript's background section, line 91-93.

**Comment 3:** Consistent and logical word order for key phrases would be easier on the reader’s eye and brain. For instance, the manuscript switches between “absenteeism and presenteeism” and “presenteeism and absenteeism”. A conventional alphabetical strategy could be used (a before p), alternatively the phenomenon which “showed up’ most strongly in the findings could be put first (from lines 597-598 it seems that presenteeism was generally more visible – at least in those studies which provided definitions). Also be consistent in how past, present and future tense is used – for instance, describe your methodology – what you did – using past tense and when reporting the findings of the reviewed studies and referring to the broader literature, settle on either past tense (“as Smith 2007 noted...”) or present tense (“as Smith 2007 note...). Also be consistent in how you refer to the studies – Table 2 uses “... et al” but in the body of the manuscript you sometimes refer to “... et al” and sometimes to “... and colleagues”.

**Response to Comment 3:** Thank you for your feedback. We have revised the logical word order for presenteeism and absenteeism and ensured that the entire manuscript is now consistently in the past tense where applicable. Additionally, we have ensured that the way we are referencing studies is consistent both in Table 2 and throughout the entire manuscript.

**Comment 4:** Terms should be carefully chosen – for instance, avoid using “concept” (lines 153-154) given that this word is already present in the abbreviation PCC. Rather use something like “construct”, “component” or “principle”. Also, be consistent – for instance, you mostly refer to the items you reviewed as “studies”, but on your PRISMA-ScR diagram you refer to “reports” and in line 240 you use the term “articles”. In lines 302-304 you use “three major topics” – you could simply say “we identified three types of antecedents, namely organisational, environmental and individual”. Both in the abstract and later in the manuscript, also make it clear how you define home environment and personal environment, if you view these as different from (or overlapping with) the work environment.

**Response to Comment 4:** Thank you for your feedback. We have reviewed the terms used and modified the PRISMA diagram to stay consistent. Thank you for pointing out about home and personal environments. In this care, there should have been just work and home environments, therefore, the misunderstanding has been resolved.

**Comment 5:** Reduce wordiness by removing superfluous words. For instances, in line 162 replace “noted how this will potentially...” (5 words) with “noted would potentially...” (3 words). There are also places where you could collapse two sentences into one – eg: lines 434-435 and 435-438. Shorten sentences where possible and avoid meaningless terms like “and so on” – see for instance lines 304-305 where you could trim the first sentence down to “Organizational antecedents included factors such as policies, job demands and workplace compensation”. Similarly, your sentence on lines 550-551, “Previous studies have investigated the antecedents of presenteeism and absenteeism for workers which have yielded similar results to this review” (20 words) could be shortened to “Previous studies investigating antecedents of absenteeism and presentations yielded similar results to our review” (14 words).

**Response to Comment 5:** That you for your comment. We have reviewed your suggestions thoroughly and implemented them into the manuscript.

**Comment 6:** Timeframe your review precisely and unambiguously. Referring to “between 2000-2023” in line 161 and “studies between the years 2019 to 2023” in line 164 is contradictory and confusing. Rather say “studies published from 2000 to 2023 (inclusive)” or, even better, “studies published from January 2019 to December 2023 (inclusive)”. Also make absolutely clear that the inclusion criterion was the publication date of the study, not the data collection date.

**Response to Comment 6:** Thank you for the comment. We have now made it explicit that the inclusion criteria was the publication date of the studies, not the data collection date. We have also revised the timeframe per your suggestion, as follows: “studies published from January 2019 to December 2023 (inclusive)”

**Comment 7:** The Methodology section is rather confusing, clumsy and circular, making it hard for the reader to picture how (and why) you went about your study. For instance, there is no explanation of why you included gender breakdowns but not age breakdowns. There is also little information on what analytical methods were used – it would be helpful to add a sentence like “Simple numerical counts and percentages were utilised to explain the studies’ geographic settings, methodologies, sample sizes and (where available) gender breakdowns. Frequency counts revealed the prevalence of presenteeism and absenteeism definitions, respectively”.

**Response to Comment 7:** For your point *“For instance, there is no explanation of why you included gender breakdowns but not age breakdowns.”* We would like to note the inclusion criteria includes studies demographic population was individuals aged 18-65 and currently employed, and that older adults and children were explicitly excluded from the study. Please let me know if you have any further comments.

Regarding the analytical methods used, we have incorporated your feedback and suggested statement.

**Comment 8:** It is a little difficult to understand the roles of the research team members, particularly in relation to BNK – described in line 177 as “an occupational therapy expert” but in line 196 as “an expert in occupational health” (occupational therapy and occupational health being two different disciplines). Also, talking about “consulting an expert” (pg 36) creates a sense of having obtained external input when instead the consultation process seems to have been internal, drawing on knowledge within the research team. External consultations may be used to add trustworthiness and credibility, but would not merit author status – instead being reflected in the Acknowledgements section.

**Response to Comment 8:** Thank you for the comment. The author BNK is an occupational therapist and a researcher in occupational health. The author is internal, not externally sourced, and is the principal investigator of this paper. We have changed the wording to occupational health expert. The author YL is also part of the internal team.

**Comment 9:** Keep your language as crisp and clear as possible when outlining your inclusion and exclusion criteria. Your last inclusion criterion in Table 1 (“Studies that implemented telework...”) should probably instead be “Studies that examined telework....”, as presumably the researchers did not implement telework themselves but instead researched how it was implemented by others. Likewise, your last exclusion criterion in Table 1 should probably read “Studies which examined in-person work environments and/or telework implemented before 2019”, to match the body of the text (lines 148-149).

**Response to Comment 9:** Thank you for the suggestion. Your recommendations have been incorporated in table 1 and throughout the manuscript.

**Comment 10:** In general, there is some muddling of the Results section (which should simply lay out the findings) and Discussion section (which should pull the findings together and make meaning out of them). Giveaways that one has veered into discussion within the results section are words like “interestingly”, “strikingly”, “It is plausible to suggest that...” or “...should be further explored in future studies”. Line 223 in the results section (“gaps were identified in definitions”) can also be deferred to the discussion. In the discussion section itself, it would be helpful to quantify the findings – eg: by mentioning the actual number (and percentage) of studies which reported a particular finding, which included a particular definition, etcetera. Also be very clear with regards to when you are mentioning the reviewed studies and when you are referring to the broader (non-reviewed) literature. In lines 344-347 it makes no sense to use the term “previous studies” in referring to other studies which were published within the same timeframe as the reviewed studies – find another way to describe them.

**Response to Comment 10:** Thank you for the suggestion, we have revised accordingly.

**Comment 11:** I find your PRISMA diagram at odds with the text – please re-check it carefully. At the first step, your diagram shows 1,117 records retrieved but in the text you refer to 1,116, so from then onwards the diagram and text do not match. You say you ultimately reviewed 21 studies but doing the maths through the PRISMA diagram suggests that there were 25 studies identified for inclusion (146-121=25); even given that three were belatedly removed, that would still leave 22, not 21? Yet your Table 2 clearly shows that only 21 studies were analysed. Please re-check all your figures for each step of the diagram. With regards to the three studies which were removed only after data extraction took place (lines 229-232), why was this so noteworthy? This kind of thing does happen during systematic reviews and once team consensus is reached that a study is not eligible after all it simply moves to the box showing all excluded studies and the grounds for exclusion.

**Response to Comment 11:**

1) Thank you for your attention to detail between the 1117 records and 1116 records. This has been resolved. 1117 articles includes 1 grey literature, so it was not from a systematic database search. 1116 articles does not include the grey literature. We have now emphasized that 1116 articles were obtained from database search, with one additional study obtained from grey literature.

2) We’d like to note that based on the PRISMA diagram, it would be 145-121, *not* 146-121. So based on the former, 145-121=24, with 3 studies belatedly removed. That would leave 21, not 22. You are correct that only 21 studies were analysed as stated in table 2.

3) with regards to the three studies that were removed after data extraction, this was noteworthy. We included your suggestion of moving to the box showing all excluded studies and the grounds for exclusion

**Comment 12:** Your including a study based on secondary data (Navarro et al) is confusing given your inclusion criterion of empirical studies only. Should that study not have been excluded on grounds of “wrong study design”? If it contained useful information such as definitions of presenteeism and/or absenteeism, this could feature in the discussion section where you relate your findings to the broader literature.

**Response to Comment 12:** Thank you for your comment. We deemed the Navarro et al study as empirical seeing how it systematically analyzed existing data to answer a new research question. We removed secondary data analysis from the chart to avoid confusion and clarified in our inclusion criteria that the empirical studies may use either primary or secondary data.

**Comment 13:** With regards to the Results section, sub-heading 3.1 (Descriptive Findings) could be replaced with “Search Results”. Section 3.2 (Study Characteristics) could give a much clearer descriptive breakdown of the studies than it currently does. Make sure that this ties in clearly with Table 2, after first resolving exactly how many studies were analysed (ie. whether Navarro et al should have been included) and then determining the best way to organise the table. Logical approaches are to list the studies in chronological order, to list them alphabetically, or to cluster them by methodologies and, within each cluster, using sub-ordering by chronology or alphabet. The accompanying text should provide additional information for readers to understand the results, with percentages added to absolute numbers to bring the results more alive and again using logical sub-ordering. For instance [using what is currently shown in your Table 2]:

• You could describe the geographic characteristics of the studies like this:

“Only 2 (9.5%) of the studies had a multi-country focus (one being focused on 28 European countries and the other having a global focus), with the other 19 (90.5%) having a single-country focus. Amongst the latter, the most represented countries were the United Kingdom (4 studies) followed by Germany, Japan and the United States (2 studies each). The remaining countries, each represented by a single study, were Austria, Canada, Lithuania, Norway, Portugal, Romania, South Korea and Spain. In all, the North American and European regions were dominant, accounting for 20 (95.2%) of the studies.”
• You could describe the methodological characteristics of the studies like this:
“From a methodological point of view, most of the studies (16; 76.2%) were quantitative, with 9 (56.3%) of those being cross-sectional, 3 (18.8%) being cohort studies, one (6.3%) each being, respectively, a secondary data analysis, a daily diary study, an experimental study and a study which did not specify what precise method was used. Three (14.3%) of the studies were qualitative, with 2 (66.7%) of these involving semi-structured interviews and one (33.3%) not specifying the precise method used. The remaining two studies (9.5%) were mixed-method studies.
• You attempted to describe the gender breakdown of the participants across all the
studies but this is not very clear – for instance, why is it “important to note that Raisiene and colleagues (2023), a Lithuanian study, had a sample comprising only 26.2% female”? And why do you include studies with 50% females amongst those “with a female minority”? You could perhaps say something like this:
“Most of the studies (18; 85.7%) provided gender breakdowns, with female representation ranging from 26.2% to 95.8% with a median value of ....%. However, of the total participant set of 54,457 across all the study samples, no gender was specified for 14,058 (25.8%)”.

**Response to Comment 13:** Thank you for the detailed and thorough feedback, we have revised the manuscript to incorporate your suggestions.

**Comment 14:** Be careful about ascribing cause where none may exist – thus, instead of saying “to identify antecedents for why...” (line 301) (my emphasis) it would be safer to say “antecedents to the occurrence of...”. As I understand it, an antecedent is simply something that existed or happened before another thing and is not always the cause or origin of the other thing, even though the two things have some logical relationship. In lines 348 and 357 you use the term “contributed to...” – did the reviewed studies use that term? Or did they just find an apparent association between two phenomena, without assigned causality? In lines 387-392 it is unclear whether the review authors are reporting their own views or reporting on the findings of the reviewed studies.

**Response to Comment 14:** Thank you for your suggestions, we have revised the manuscript accordingly.

**Comment 15:** In the Discussion section I suggest replacing the terms “Western bias” and “non-western countries” when discussing the geographical limitations of the review. Rather use more contemporary terms like Global North and Global South, or refer to most of the studies having been carried out in High-Income Countries rather than Low and Middle Income Countries. It is important to make clear what your own recommendations are, emanating from your study, rather than just referring to recommendations made by other authors. The Conclusion section should be far shorter, summing up the essence of the review in a few sentences rather than repeating aspects of the discussion and recommendations. The very last sentence, in using the phrase “...for teleworkers and non-teleworkers alike” (line 670), actually seems to dilute the whole purpose of the review and leave the reader with a sense of incompletion.

**Response to Comment 15:** Thank you for the comment. We have replaced the terms “Western bias” and “non-western countries” when discussing the geographic limitations of the review. We have also shortened the conclusion section. Finally, we have removed the “teleworkers and non-teleworkers alike” to avoid confusion for the reader.

**Comment 16:** Finally, with regards to proofreading and error correction, here are some pointers:
a. Whilst re-checking and finalising Table 2 pay attention to fine points like formatting – for instance, under the Sample Size column you have 275 next to Biron et al, rather than n=275. You also use the format n=x in some places and n = x in others.
b. Watch out for these typographical and grammatical errors:
• Word omissions – eg: the word “to” seems to have been left out on line 79 (“...due [to] working while sick”).
• Incorrect grammar – eg: “physical present” on lines 80 and 81 should read “physically present”; “being physically absent to work” in line 82 should probably read “being physically absent from work”; “our eligibility criteria was modified” in line 160 should read “our eligibility criteria were modified”; “Data charting and data reporting was performed” in line 181 should read “Data charting and data reporting were performed”.
c. Missing or inconsistent punctuation and numbering – lack of full stops in various places; different ways of using the abbreviation of ‘for example” (shown as e.g. in some places and e.g., in others); a missing apostrophe in line 104 (“...workers’ ill-health”); missing question marks for the research questions on lines 105-107; missing comma in line 127; the sub-heading “Data Synthesis” should be numbered “2.5 Data Synthesis”.
• Misspelling of significant words - eg: ‘PRIMA-ScR’ instead of ‘PRISMA-ScR’ in line 120; ‘preformed’ instead of ‘performed’ in line 370; ‘presentism’ instead of ‘presenteeism’ in line 640.
• Strange symbols appearing in ref 9 on the reference list.
• Clumsy wording – eg: a sentence in lines 150-151 would be shorter and read better if worded “...we wanted to ensure that organizations that implemented telework did so due to COVID-19”.
d. Avoid repetition and wordiness, for example:
• The word “overall” is used to start two close sentences on page 2 (lines 15 and 19).
• In lines 47 and 48 there appears “...after the height of the COVID-19 pandemic restrictions-COVID-19 restrictions...”.
e. Referencing errors and inconsistencies need to be identified and corrected, for example:
• ‘Tremblay’ is shown as a source in line 54 but does not appear in the reference list.
• Inconsistent referencing in relation to Ruhle makes it difficult to know whether the author cited in line 60 was actually Ruhle & Schmoll (no. 18 on the reference list) or Ruhle as shown in reference 16 on the reference list).
• Single author Grigore (ref 23 on the reference list) is referred to as “Grigore and colleagues” in various places in the body of the manuscript. Reference 23 also seems incomplete, ending with “8:”. On line 293 you need to add [23] after mentioning Grigore and before citing Cooper and Locke.
• In lines 417-418 you refer to Brosi and colleagues, but ref 41 shows only two authors, Brosi & Gerpott.
• Different dates shown for Shafer and colleagues – shown as 2023 in lines 453-454 but Table 2 (pg 16) and ref 17 gives the date as 2020.
• There is a mismatch in dates for Arksey & O’Malley – given as 2m007 in the body of the manuscript (lines 113, 170 and 182) but as 2005 in ref 28.
• There is incorrect spelling of Laranjeira/Laranjira, between the text and the reference list.
• The verbatim quote from Cooper & Locke in lines 293-295 should get a page number if possible.
• In line 171 you mention Zotero but you provide no reference for this, in the way you have done for Covidence.

**Response to Comment 16:** Thank you for the detailed comment. We have reviewed all your points to improve the grammar and structure of the manuscript.

We would like to thank the reviewers for your helpful feedback and comments. We believe their suggestions have improved the quality of our manuscript.
