# Supplementary material for: Sick leave or work sick? Examining the antecedents and conceptualizations of presenteeism and absenteeism among teleworkers during COVID-19: A scoping review
Source: PLOS Ment Health. 2025 May 13;2(5):e0000300. doi: 10.1371/journal.pmen.0000300 (PMC12798164; doi:10.1371/journal.pmen.0000300)
Supplement: Attachment — Submitted filename: Excluded Studies .docx [file pmen.0000300.s005.docx]

**Table 1:** Included Articles

|  | **Article** | **Date of Data Collection** |
| --- | --- | --- |
| 1 | Adisa 2023 | July and September 2020 |
| 2 | Biron 2021 | April, June, December 2020 |
| 3 | Borge 2023 | October 2019 – March 2020 |
| 4 | Brosi 2023 | Received February 2020 |
| 5 | Gerich 2022 | February and March 2021 |
| 6 | Grigore 2020 | July 2020 |
| 7 | Hadi 2020 | April 2020 |
| 8 | Keightley 2023 | April to May 2020 |
| 9 | Mauricio and Laranjeira 2023 | September 2022 to October 2022 |
| 10 | Michael 2021*dissertation* | March 31, 2021 (date published) |
| 11 | Okawara 2023 | December 2020 |
| 12 | Raisiene 2023 | Doesn’t say date only that it was during the pandemic shutdown (Received September 2022) |
| 13 | Ruhle and Schmoll 2021 | September and October 2020 |
| 14 | Ryoo 2023 | Oct 2020- Dec 2020 & Jan 2021 – Apr 2021 |
| 15 | Shafer 2023 | Mar 2020 – Nov 2020 |
| 16 | Takayama 2023 | August – September 2020; follow-up survey Sept – Oct 2021 and Feb 2022 |
| 17 | Walker 2023 | Sept and Oct 2021 |
| 18 | Yildirim 2022 | March 2020 |

**Table 2:** Excluded Articles

| **Count** | **Article** | **Reason for Exclusion** | **Study Number** | **Author/Year** |
| --- | --- | --- | --- | --- |
|  | **Title & Abstract Screening** | | | |
| 1 | Sickness Presenteeism in the Aftermath of COVID-19: Is Presenteeism Remote-Work Behavior the New (Ab)normal?. | Does not meet inclusion criteria | 79 | Ferreira 2022 |
| 2 | Employees' dedication to working from home in times of COVID-19 crisis | Does not meet inclusion criteria | 2539 | Prodanova 2022 |
| 3 | Techno-Stress: Damage Caused by New Emerging Risks | Does not meet inclusion criteria | 2644 | RubénRodríguezElizalde 2021 |
| 4 | MANAGERIAL & ORGANIZATIONAL COGNITION Conference Paper Abstracts. | Does not meet inclusion criteria | 233 | N/A |
| 5 | WORKER ATTITUDES AND INDUSTRIAL ABSENTEEISM: A STATISTICAl APPRAISAL. | Does not meet inclusion criteria | 189 | Noland 1945 |
| 6 | Circuits Split on Definition of a "Disability-Related Injury.". | Does not meet inclusion criteria | 191 | Lavin 2012 |
| 7 | Sixth Circuit rules that telecommuting may be a reasonable accommodation. | Does not meet inclusion criteria | 145 | LaPlante 2014 |
| 8 | Power of Love (AGAPE) in Leadership: A Theoretical Model and Research Agenda | Does not meet inclusion criteria | 2694 | Carvalho 2020 |
| 9 | EFFECT OF WORK FROM HOME ON WORKPLACE ROMANCE: A SOCIO-ECONOMIC PERSPECTIVE | Does not meet inclusion criteria | 2767 | Mishra 2021 |
| 10 | The Economic Impact of Lockdowns: A Persistent Inoperability Input-Output Approach | Does not meet inclusion criteria | 2643 | Yu et al. 2020 |
| 11 | Risk of Increased Acceptance for Organizational Nepotism and Cronyism during the COVID-19 Pandemic | Does not meet inclusion criteria | 2670 | Ignatowski 2021 |
| 12 | Severe Staffing And Personal Protective Equipment Shortages Faced By Nursing Homes During The COVID-19 Pandemic | Does not meet inclusion criteria | 2593 | McGarry 2020 |
| 13 | Does distribution of menstrual products through community-based, peer-led sexual and reproductive health services increase use of appropriate menstrual products? Findings from the Yathu Yathu trial | Does not meet inclusion criteria | 2211 | Hensen 2023 |
| 14 | How costly is the first prenatal clinic visit? Analysis of out-of-pocket expenditure in rural Sri Lanka - a country with free maternal health care | Does not meet inclusion criteria | 2762 | Sajaan 2021 |
| 15 | Rural Aging during COVID-19: A Case Study of Older Voluntarism | Does not meet inclusion criteria | 2459 | Colibaba 2021 |
| 16 | Anxious hotel employees in China: Engaged or exhausted? Multiple effects of workplace anxiety | Does not meet inclusion criteria | 2294 | Yin 2023 |
| 17 | Corporate social purpose statements and employee perceptions about the CEO and the corporation: A large sample natural experiment | Does not meet inclusion criteria | 2221 | Lianidou 2023 |
| 18 | Work-family research: A review and next steps | Does not meet inclusion criteria | 2293 | Allen 2023 |
| 19 | Losing compassion for patients? The implications of COVID-19 on compassion fatigue and event-related post-traumatic stress disorder in nurses | Does not meet inclusion criteria | 2451 | Hochwarter 2022 |
| 20 | The Effects of Employee Ownership on Organizational Commitment and Job Satisfaction: An Empirical Evidence From French Listed Companies | Does not meet inclusion criteria | 2765 | Elouadi 2020 |
| 21 | Organizational change and job insecurity: the moderating role of employability | Does not meet inclusion criteria | 2693 | Çalışkan 2020 |
| 22 | Home-based work, time allocations, and subjective well-being: gender differences in the United Kingdom | Does not meet inclusion criteria | 2291 | Giménez-Nadal 2023 |
| 23 | Changing Face of Job Satisfaction of IT Sector Employees in view of Covid-19 in India | Does not meet inclusion criteria | 2406 | Gupta 2023 |
| 24 | Benefits of Flexible Working System During Covid-19 Pandemic: A Field Study in Turkey | Does not meet inclusion criteria | 2498 | Kaya 2022 |
| 25 | Professional correlates of insomnia. | Does not meet inclusion criteria | 97 | Leger 2006 |
| 26 | Work engagement and employee satisfaction in the practice of sustainable human resource management – based on the study of Polish employees | Does not meet inclusion criteria | 2121 | Sypniewska 2023 |
| 27 | The experiences and perceptions of wellbeing provision among English ambulance services staff: a multi-method qualitative study | Does not meet inclusion criteria | 2326 | Viet-HaiPhung 2022 |
| 28 | Psychosocial burdens in palliative care – a longitudinal cohort study in nursing homes and impacts of the COVID-19 pandemic | Does not meet inclusion criteria | 2107 | Bußmann 2023 |
| 29 | Burnout in Virginia's Community College Adjuncts With Relation to Gender, Age, and Number of Jobs | Does not meet inclusion criteria | 265 | Stowe 2023 |
| 30 | Why and how the workplace should promote employee planning for caregiving | Does not meet inclusion criteria | 272 | Norstrand 2022 |
| 31 | On Pandemic Preparedness: How Well is the Modeling Community Prepared for COVID‑19? | Does not meet inclusion criteria | 2681 | Desai 2020 |
| 32 | Resilience of Brazilian health-care professionals during the pandemic | Does not meet inclusion criteria | 2581 | Araujo 2022 |
| 33 | COVID-19 AS A FACTOR FOR THE DIGITALISATION IN HUMAN RESOURCE MANAGEMENT IN THE BULGARIAN FINANCIAL INSTITUTIONS | Does not meet inclusion criteria | 2588 | Zahariev 2022 |
| 34 | Sustainability elements of companies that are affected by pandemics | Does not meet inclusion criteria | 2422 | Moolman 2023 |
| 35 | Back Page. | Does not meet inclusion criteria | 202 | Keinigs 2004 |
| 36 | 28th Annual Conference of the International Society for Quality of Life Research | Does not meet inclusion criteria | 2348 | Long et al., 2021 |
| 37 | Employee substitutability as a tool to improve the robustness in personnel scheduling. | Does not meet inclusion criteria | 244 | Ingels 2017 |
| 38 | Ten Years of Research on Psychosocial Risks, Health, and Performance in Latin America: A comprehensive Systematic Review and Research Agenda | Does not meet inclusion criteria | 2343 | Pujol-Cols 2021 |
| 39 | Working while sick: validation of the multidimensional presenteeism exposures and productivity survey for nurses (MPEPS-N). | Does not meet inclusion criteria | 53 | Lui 2019 |
| 40 | A cross‐sectional study of infection control measures against COVID‐19 and psychological distress among Japanese workers | Does not meet inclusion criteria | 2307 | Yasuda 2021 |
| 41 | Occupational Stress: A Review on Conceptualisations, Causes and Cure. | Does not meet inclusion criteria | 223 | Babatunde 2013 |
| 42 | Productivity Loss Across Socioeconomic Groups Among Patients With Low Back Pain or Osteoarthritis: Estimates Using the Friction-Cost Approach in Norway | Does not meet inclusion criteria | 2128 | Hansen 2023 |
| 43 | Is there a bidirectional relationship between workplace bullying and the risk of sickness absence? Systematic review and meta-Analysis of prospective studies | Does not meet inclusion criteria | 2113 | Liao 2023 |
| 44 | A cross‐sectional study of socioeconomic status and treatment interruption among Japanese workers during the COVID‐19 pandemic | Does not meet inclusion criteria | 2304 | Fujimoto 2021 |
| 45 | Curing Spring Fever. | Does not meet inclusion criteria | 156 | Demars 2007 |
| 46 | The Effect of Job Insecurity on Bank Employees’ Job Stress and Job Burnout During COVID-19: A Moderated Mediation Model | Does not meet inclusion criteria | 2357 | Peng 2022 |
| 47 | Do worksite health promotion programs (WHPP) influence presenteeism among employees? A systematic review 1 | Does not meet inclusion criteria | 6 | Støren 2023 |
| 48 | Paid Leave and Access to Telework as Work Attendance Determinants during Acute Respiratory Illness, United States, 2017-2018. | Does not meet inclusion criteria | 18 | Ahmed 2020 |
| 49 | Context and consistency in absenteeism: studying social and dispositional influences across multiple settings | Does not meet inclusion criteria | 187 | Harrison 2003 |
| 50 | Homeless population and COVID-19 | Does not meet inclusion criteria | 2515 | FreitasHonorato 2020 |
| 51 | The psychosocial work conditions and mental well-being of independent school heads in South Africa | Does not meet inclusion criteria | 2453 | Breedt 2023 |
| 52 | Human Resource Practices and Job Satisfaction in the Hotel sector in India: An Organizational Perspective with Smart PLS Analysis | Does not meet inclusion criteria | 2494 | Kumar 2022 |
| 53 | Relational demography in organizations: an assessment of the explored and overlooked | Does not meet inclusion criteria | 2488 | Kaur 2022 |
| 54 | Hope you're keeping well | Does not meet inclusion criteria | 2355 | Taylor 2020 |
| 55 | Re-examining the nexuses of communicable diseases, environmental performance, and dynamics of sustainable Development in OECD countries | Does not meet inclusion criteria | 2518 | Yu 2022 |
| 56 | The Role of Scientific Expertise in COVID-19 Policy-making: Evidence from Four European Countries | Does not meet inclusion criteria | 2547 | Hodges 2022 |
| 57 | Global tracking of access and quality in early childhood care and education | Does not meet inclusion criteria | 2220 | Raikes 2023 |
| 58 | Modelo de desempeño en la tarea: estudio empírico con PLS-SEM y mapa de importanciarendimiento | Does not meet inclusion criteria | 2447 | Cavazos-Arroyo· 2023 |
| 59 | Impacts of social and economic factors on the transmission of coronavirus disease 2019 (COVID-19) in China | Does not meet inclusion criteria | 2363 | QiuYun 2020 |
| 60 | Event-Oriented Organizational Behavior Research: A Multilevel Review and Agenda for Future Research | Does not meet inclusion criteria | 2299 | Liu 2023 |
| 61 | Impact of COVID-19 Pandemic on Information Technology in Multiple Sectors of the Economy | Does not meet inclusion criteria | 2579 | Desai 2022 |
| 62 | What the COVID-19 pandemic tells us about the need to develop resilience in the nursing workforce | Does not meet inclusion criteria | 2600 | Duncan 2020 |
| 63 | Man or machine – or something in between? Social responses to voice assistants at work and their effects on job satisfaction | Does not meet inclusion criteria | 2209 | Ossadnik 2023 |
| 64 | COVID-19 and Change | Does not meet inclusion criteria | 2746 | Goede 2021 |
| 65 | Helping school refusing children and their parents: A guide for school-based professionals. | Does not meet inclusion criteria | 92 | Kearney 2008 |
| 66 | Quality of life and severity of symptoms among patients with various degrees of reflux esophagitis: a prospective study | Does not meet inclusion criteria | 2116 | Mari 2023 |
| 67 | COVID-19 Disruption and Meaningful Work: The Mediating Role of Family–Work Conflict | Does not meet inclusion criteria | 2483 | Vara-Horna 2023 |
| 68 | Work Flexibility and Job Satisfaction: Shifting Workplace Norms | Does not meet inclusion criteria | 2384 | Andrade 2023 |
| 69 | Bridging the gap among professionals who address youths with school absenteeism: Overview and suggestions for consensus. | Does not meet inclusion criteria | 105 | Kearney 2003 |
| 70 | Absenteeism in the UK: A Comparison Across Genders. | Does not meet inclusion criteria | 181 | Bridges 2001 |
| 71 | Impact of Influenza and Influenza-Like Illness on Work Productivity Outcomes: A Systematic Literature Review. | Does not meet inclusion criteria | 216 | Zumofen 2023 |
| 72 | Do job stress, health, and presenteeism differ between Chinese healthcare workers in public and private hospitals: a cross sectional study. | Does not meet inclusion criteria | 52 | Yang 2020 |
| 73 | Worker productivity outcome measures in arthritis. | Does not meet inclusion criteria | 123 | Escorpizo 2007 |
| 74 | Is Working from Home a Blessing or a Burden? Home Demands as a Mediator of the Relationship Between Work Engagement and Work-Life Balance | Does not meet inclusion criteria | 2160 | Ugwu 2023 |
| 75 | Longitudinal development of well-being among Finnish employees during 2019–2021: Relationships with personality trait profiles | Does not meet inclusion criteria | 2287 | Oksa 2023 |
| 76 | Do elections accelerate the COVID-19 pandemic? | Does not meet inclusion criteria | 2342 | PalgutaJán 2022 |
| 77 | Is the US 3PL industry overcoming paradoxes amid the pandemic? | Does not meet inclusion criteria | 2395 | Ashraf 2022 |
| 78 | The effect of occupational commitment and mobbing on the intention to leave in road transportation during the COVID-19 pandemic period | Does not meet inclusion criteria | 2664 | Yangınlar 2021 |
| 79 | Is paid sick leave bad for business? A systematic review | Does not meet inclusion criteria | 2190 | VanderWeerdt 2023 |
| 80 | Social Justice Is Not the COVID-19 Vaccine Alone: It Is Addressing Structural Racism Through Social Policies That Shape Health | Does not meet inclusion criteria | 2352 | Mays 2021 |
| 81 | FOUR FACTORS INFLUENCING CONVERSION TO A FOUR-DAY WORK WEEK. | Does not meet inclusion criteria | 237 | Hartman 1977 |
| 82 | Getting Real: The Maryland Healthcare Ethics Committee Network’s COVID-19 Working Group Debriefs Lessons Learned | Does not meet inclusion criteria | 2710 | Norton 2021 |
| 83 | The clinical & economic burden of treatment-resistant depression in the Gulf-Cooperation Council: The Kingdom of Saudi Arabia, Kuwait and the United Arab Emirates | Does not meet inclusion criteria | 2126 | Akram 2023 |
| 84 | Does “Nei Juan” affect “Tang Ping” for hotel employees? The moderating effect of effort-reward imbalance | Does not meet inclusion criteria | 2303 | Yin 2023 |
| 85 | Economic burden of urgency urinary incontinence in the United States: a systematic review. | Does not meet inclusion criteria | 33 | Coyne 2014 |
| 86 | COVID-19 Pandemic Factors and Depressive Symptoms Among Primary Care Workers in São Paulo, Brazil, October and November 2020 | Does not meet inclusion criteria | 2457 | daSilva 2022 |
| 87 | A comparison of serum inflammatory parameters in progressive forms of multiple sclerosis | Does not meet inclusion criteria | 2233 | Nowak-Kiczmer 2023 |
| 88 | Living through uncertainty: a qualitative study on leadership and resilience in primary healthcare during COVID-19 | Does not meet inclusion criteria | 2450 | Karreinen 2023 |
| 89 | The potential of a multimodal digital care program in addressing healthcare inequities in musculoskeletal pain management | Does not meet inclusion criteria | 2108 | Areias 2023 |
| 90 | Malaysia Pharmaceuticals & Healthcare Report - Q4 2022 | Does not meet inclusion criteria | 274 | N/A |
| 91 | The effect of pay for performance on work attitudes in the private, public, and nonprofit sectors: A panel study from South Korea | Does not meet inclusion criteria | 2298 | Bae 2023 |
| 92 | Does Absence Make the Heart Grow Less Fond? Spatial Proximity Partially Predicts Family Forest Landowner Engagement | Does not meet inclusion criteria | 2228 | Caputo 2023 |
| 93 | Post COVID‐19 vaccination: AusVaxSafety survey participation and adverse events – a community‐based regional Queensland study | Does not meet inclusion criteria | 2473 | Hamilton 2022 |
| 94 | Telework in Canada : Who Is Working from Home during the COVID-19 Pandemic ? | Does not meet inclusion criteria | 2656 | Chowhan 2021 |
| 95 | THE ROLE OF RISK MANAGEMENT With COVID-19 & Its Impact on Pandemics | Does not meet inclusion criteria | 2759 | Lyon 2020 |
| 96 | COVID-19: unbalanced management of occupational risks—case of the analysis of the chemical risk related to the use of disinfectants in the dairy industry in Morocco | Does not meet inclusion criteria | 2549 | RachidiHafida 2022 |
| 97 | The influence of emotional intelligence and resilience on work engagement amongst nurses in public hospitals | Does not meet inclusion criteria | 2625 | Chikobvu 2022 |
| 98 | Healthy aging, absenteeism, and presenteeism. | Does not meet inclusion criteria | 63 | Truxillo 2022 |
| 99 | Supervisor Support, Self-Efficacy, and Employee Performance: The Mediating Role of Office De-Clutter | Does not meet inclusion criteria | 2325 | Saleem 2022 |
| 100 | Effects of Healthcare Organization Actions and Policies Related to COVID-19 on Perceived Organizational Support Among U.S. Internists: A National Study | Does not meet inclusion criteria | 2400 | Sonis 2022 |
| 101 | Outcomes of rapid digital transformation of large-scale communications during the COVID-19 pandemic | Does not meet inclusion criteria | 2583 | Strong 2021 |
| 102 | Lessons from the COVID epicenter: how teacher candidates of color and the academy adapted to remote instruction | Does not meet inclusion criteria | 2618 | Magaldi 2022 |
| 103 | The effect of work motivation, work environment, and job satisfaction on organizational citizenship behavior and their impact on employees performance of RSU Menteng Mitra Afia during the Covid-19 pandemic | Does not meet inclusion criteria | 2726 | Rinaldi 2021 |
| 104 | The New Normal for Health Care Providers (HCPs) and Allied Personnel in Pharmaceutical Industry in Post-Pandemic Era | Does not meet inclusion criteria | 2755 | Chattopadhyay 2021 |
| 105 | Effects of a clinical decision support system and patient portal for preventing medication-related falls in older fallers: Protocol of a cluster randomized controlled trial with embedded process and economic evaluations (ADFICE_IT) | Does not meet inclusion criteria | 2118 | deWildt 2023 |
| 106 | The hard truth about telecommuting. | Does not meet inclusion criteria | 151 | Noonan 2012 |
| 107 | Employee Compensation and Benefits Pre and Post COVID-19 | Does not meet inclusion criteria | 2501 | Shtembari 2022 |
| 108 | Telecommute not always ADA accommodation. (cover story) | Does not meet inclusion criteria | 176 | N/A |
| 109 | The Effect of Presenteeism on Productivity Loss in Nurses: The Mediation of Health and the Moderation of General Self-Efficacy. | Does not meet inclusion criteria | 31 | Li 2019 |
| 110 | Pros and cons of telework. | Does not meet inclusion criteria | 154 | N/A |
| 111 | South African Energy Minister Has Covid-19 Amid Power Crisis. | Does not meet inclusion criteria | 157 | Burkhardt 2020 |
| 112 | The influence of organizational responses to the COVID-19 pandemic on employee outcomes | Does not meet inclusion criteria | 2685 | Shepard 2021 |
| 113 | From 60 to 0: Denver group halts turnover at area businesses through telework. | Does not meet inclusion criteria | 139 | Silva 2007 |
| 114 | COVID-19 and employee psychological safety: Exploring the role of signaling theory | Does not meet inclusion criteria | 2649 | Green 2021 |
| 115 | Focused Time Saves Nine: Evaluating Computer-Assisted Protected Time for Hybrid Information Work | Does not meet inclusion criteria | 2265 | DasSwain 2023 |
| 116 | A Health-Impact Assessment of an Ergonomic Measure to Reduce the Risk of Work-Related Lower Back Pain, Lumbosacral Radicular Syndrome and Knee Osteoarthritis among Floor Layers in The Netherlands | Does not meet inclusion criteria | 2288 | Kuijer 2023 |
| 117 | A Rapid Qualitative Appraisal of the Impact of COVID-19 on Long-term Care Communities in the United States: Perspectives from Area Aging Staff and Advocates | Does not meet inclusion criteria | 2310 | Freidus 2020 |
| 118 | Prevalence of work-related musculoskeletal disorders among workers in the automobile manufacturing industry in China: a systematic review and meta-analysis | Does not meet inclusion criteria | 2231 | He 2023 |
| 119 | Workplace health promotion programs among health workers: a systematic literature review | Does not meet inclusion criteria | 2149 | González 2023 |
| 120 | How Cal Newport rewrote the productivity gospel | Does not meet inclusion criteria | 263 | Weaver 2023 |
| 121 | Absence management. | Does not meet inclusion criteria | 208 | N/A |
| 122 | [Irritable bowel syndrome: epidemiology/economic burden]. | Does not meet inclusion criteria | 40 | Dapoigny 2009 |
| 123 | Predicting On-the-Job Substance Abuse With a Written Integrity Test. | Does not meet inclusion criteria | 247 | Mastrangelo 2001 |
| 124 | TEACHERS' AND PROFESSORS' PERCEPTION OF TELEWORK IN ROMANIA | Does not meet inclusion criteria | 2701 | Sârbu 2021 |
| 125 | LINK BETWEEN LEADERSHIP STYLES OF MANAGERS AND EMPLOYEE`S PERFORMANCE IN ORGANIZATIONS DURING COVID-19 CRISIS | Does not meet inclusion criteria | 2561 | CUHADAR 2022 |
| 126 | The impact of COVID-19 on service delivery systems: evidence from a survey of United States refugee resettlement agencies | Does not meet inclusion criteria | 2442 | Moise 2022 |
| 127 | With Telework Week underway, timely study confirms employee and employer benefits. | Does not meet inclusion criteria | 153 | Weldon 2014 |
| 128 | The relationship between immune fitness and saliva biomarkers of systemic inflammation | Does not meet inclusion criteria | 2132 | Mulder 2023 |
| 129 | Structural Obstacles for Women in Academia: Availability and Costs of Campus Child Care | Does not meet inclusion criteria | 2706 | Dolamore 2021 |
| 130 | Flexible labour policies as competitive advantage | Does not meet inclusion criteria | 2279 | Antunes 2023 |
| 131 | Assessing the impact of a knowledge translation intervention on physical therapists’ self-efficacy and implementation of motor learning practice | Does not meet inclusion criteria | 2236 | Kafri 2023 |
| 132 | RISK ANALYSIS OF CONSTRUCTION A LOGISTIC CENTRE IN ECONOMIC AND ENVIRONMENTAL ASPECTS. | Does not meet inclusion criteria | 182 | Tokarski 2022 |
| 133 | MEASUREMENT AND MEANING OF JOB SATISFACTION. | Does not meet inclusion criteria | 238 | Wanous 1972 |
| 134 | Teaching and learning “Digital Wellbeing” | Does not meet inclusion criteria | 2224 | Monge Roffarello 2023 |
| 135 | The impact of COVID-19 on psychiatric and mental health services in Europe: suffering experienced by professionals | Does not meet inclusion criteria | 2570 | Kane 2022 |
| 136 | Understanding the Global Burden of Influenza in Adults Aged 18–64 years: A Systematic Literature Review from 2012 to 2022 | Does not meet inclusion criteria | 2290 | Maleki 2023 |
| 137 | Supply chain risk mitigation strategies during COVID-19: exploratory cases of “make-to-order” handloom saree apparel industries | Does not meet inclusion criteria | 2578 | Dohale 2022 |
| 138 | Promoting meaningfulness in work for higher job satisfaction: will intent to quit make trouble for business managers? | Does not meet inclusion criteria | 2109 | Haque 2023 |
| 139 | RESOURCE PERCEPTIONS AND EMPLOYEE WELL-BEING DURING THE COVTD-19 PANDEMIC: THE ROLE OF PERSONAL CONTROL AS A MODERATOR | Does not meet inclusion criteria | 2673 | Jha 2022 |
| 140 | THE PROBLEM OF NEW NON-STANDARD EMPLOYMENT FORMS: ANALYSIS OF CHALLENGES AND OPPORTUNITIES | Does not meet inclusion criteria | 2436 | Kolomoets 2023 |
| 141 | The evolving international entrepreneurship orientations and international entrepreneurship capital in the rapidly changing and digitizing international environments | Does not meet inclusion criteria | 2590 | Etemad 2022 |
| 142 | Digital Leadership in an Ever-Changing World: A Bibliometric Analysis of Trends and Challenges | Does not meet inclusion criteria | 2143 | Espina-Romero 2023 |
| 143 | Linking transformational leadership with job satisfaction: the mediating roles of trust and team cohesiveness | Does not meet inclusion criteria | 2607 | Siswanto 2022 |
| 144 | Employee financial health comes to the fore after COVID | Does not meet inclusion criteria | 2553 | Jackson 2022 |
| 145 | Hard day's work: A boon for performance but a bane for satisfaction? | Does not meet inclusion criteria | 99 | McFarlin 2006 |
| 146 | “Back pain is part of sport … I'm just gonna have to live with it”: Exploring the lived experience of sport-related low back pain in adolescent athletes | Does not meet inclusion criteria | 2142 | Wall 2023 |
| 147 | In search of an international multidimensional action plan for second victim support: a narrative review | Does not meet inclusion criteria | 2244 | Seys 2023 |
| 148 | HUMAN RESOURCES Conference Paper Abstracts. | Does not meet inclusion criteria | 168 | N/A |
| 149 | CONSIDERATIONS CONCERNING THE IMPLICATIONS OF THE COVID 19 PANDEMIC ON THE ROLE OF THE CORPORATE GOVERNANCE IN THE ENSURANCE OF THE CONTINUITY OF THE ACTIVITY | Does not meet inclusion criteria | 2751 | Socoliuc 2020 |
| 150 | sick leave. | Does not meet inclusion criteria | 199 | N/A |
| 151 | Time Reallocation and the Cost and Benefit of School Closures during an Epidemic | Does not meet inclusion criteria | 2641 | Bayham 2021 |
| 152 | Management practices impacting on the rostering of medical scientists in the Australian healthcare sector | Does not meet inclusion criteria | 2397 | Cavanagh 2022 |
| 153 | Glass ceiling required. | Does not meet inclusion criteria | 218 | Roberts 2003 |
| 154 | Health system impacts of SARS-CoV − 2 variants of concern: a rapid review | Does not meet inclusion criteria | 2678 | Dol 2022 |
| 155 | ROI DE RR.HH. | Does not meet inclusion criteria | 165 | Ibáñez 2013 |
| 156 | Academic Stress Mediates the Relationship Between Satisfaction with Travel and Psychological Well-being | Does not meet inclusion criteria | 2734 | Mallari 2021 |
| 157 | Bullying at Work and Its Impact on Job Satisfaction: An Exploration of School Psychologists | Does not meet inclusion criteria | 2543 | Crothers 2022 |
| 158 | Health care provider time in public primary care facilities in Lima, Peru: a cross-sectional time motion study | Does not meet inclusion criteria | 2657 | Leslie 2021 |
| 159 | Telework and women: perceptions of work and family life balance | Does not meet inclusion criteria | 2418 | DeAguiar 2022 |
| 160 | Contemporary Hermits: A Developmental Psychopathology Account of Extreme Social Withdrawal (Hikikomori) in Young People | Does not meet inclusion criteria | 2297 | Muris 2023 |
| 161 | La conciliación que viene y su visión desde la comunicación interna. | Does not meet inclusion criteria | 133 | Cid 2021 |
| 162 | Effects of COVID-19 in the Financial Statements of Selected Companies Listed in Warsaw Stock Exchange | Does not meet inclusion criteria | 2753 | Hońko 2020 |
| 163 | Stressing the importance of mental health in I-O courses | Does not meet inclusion criteria | 2757 | Mendoza 2020 |
| 164 | Monetary valuation of COVID-19 informal care: caregivers’ willingness to pay and willingness to accept | Does not meet inclusion criteria | 2421 | Ramezani-Doroh 2023 |
| 165 | Case study of a stepped-care psychological service for healthcare professionals working in critical care | Does not meet inclusion criteria | 2766 | Galati 2021 |
| 166 | “I stretch them out as long as possible:” U.S. women’s experiences of menstrual product insecurity during the COVID-19 pandemic | Does not meet inclusion criteria | 2213 | Schmitt 2023 |
| 167 | Unequal access? Use of sickness absence benefits by precariously employed workers with common mental disorders: A register-based cohort study in Sweden | Does not meet inclusion criteria | 2119 | Hernando-Rodriguez 2023 |
| 168 | Social Inclusion of older adults During COVID 19 pandemic | Does not meet inclusion criteria | 2596 | Subudhi 2020 |
| 169 | Effects of Covid-19 Related Government Budget Shortfall on Funding and Provision of Essential Public Services | Does not meet inclusion criteria | 2750 | Aikins 2021 |
| 170 | Experts warn Elon Musk that Tesla's tough return to office policy could 'backfire'. | Does not meet inclusion criteria | 174 | N/A |
| 171 | AN EMPIRICAL EXAMINATION OF SEVERAL JOB ATTRACTION MEASURES. | Does not meet inclusion criteria | 231 | Barth 1973 |
| 172 | When does presenteeism harm productivity the most? Employee motives as a key moderator of the presenteeism–productivity relationship | Does not meet inclusion criteria | 275 | Henderson 2022 |
| 173 | Work-related musculoskeletal disorders are associated with impaired presenteeism in allied health care professionals. | Does not meet inclusion criteria | 35 | Campo 2012 |
| 174 | Enhancing the role of facilities management in the fight against the COVID-19 (SARS-CoV-2) pandemic in developing countries’ public hospitals | Does not meet inclusion criteria | 2655 | Amos 2021 |
| 175 | Does social distancing make the heart grow fonder? The impact of work spouse interaction frequency on organizational outcomes | Does not meet inclusion criteria | 2259 | Frieder 2023 |
| 176 | Impact of teleworking on job satisfaction among Slovakian employees in the era of COVID-19 | Does not meet inclusion criteria | 2636 | Karácsony 2021 |
| 177 | Exploring the concept of presenteeism in nursing: A hybrid concept analysis | Does not meet inclusion criteria | 1 | Mohammadi 2021 |
| 178 | A cross‐sectional study on perceived workplace health support and health‐related quality of life | Does not meet inclusion criteria | 2381 | Kurogi 2021 |
| 179 | Give Me Five: The Most Important Social Values for Well-Being at Work | Does not meet inclusion criteria | 2580 | Reinaldo Sousa Santos 2022 |
| 180 | Resistance of commercial banks to the crisis caused by the COVID-19 pandemic: the case of Poland | Does not meet inclusion criteria | 2599 | Korzeb 2020 |
| 181 | Risk Perception In Practice Of Polish Mncs In Kazakhstan During Covid-19 Time | Does not meet inclusion criteria | 2524 | Jaworek 2022 |
| 182 | Cultural Wellbeing Index: A Dynamic Cultural Analytics Process for Measuring and Managing Organizational Inclusion as an Antecedent Condition of Employee Wellbeing and Innovation Capacity | Does not meet inclusion criteria | 2713 | Lister 2021 |
| 183 | E-work evaluation through work–life balance, job effectiveness, organizational trust and flexibility: evidence from Kosovo during COVID-19 | Does not meet inclusion criteria | 2526 | Gashi 2022 |
| 184 | Teleworking in Manufacturing: Dealing with the Post-Pandemic COVID-19 Challenge | Does not meet inclusion criteria | 2199 | Fontaneda 2023 |
| 185 | Millennials in the workplace: perceived supervisor support, work–life balance and employee well–being | Does not meet inclusion criteria | 2638 | Nabawanuka 2022 |
| 186 | Workplace deviance in the virtual workspace | Does not meet inclusion criteria | 2727 | Shrivastava 2021 |
| 187 | How workload impacts the employee performance and how work stress acts as a mediating variable in shoes manufacturing company | Does not meet inclusion criteria | 2430 | Herdiana 2023 |
| 188 | The impact of epidemics on agricultural production and forecast of COVID-19 | Does not meet inclusion criteria | 2602 | Zhang 2020 |
| 189 | Employees’ preferences on organisational aspects of psychotherapeutic consultation at work by occupational area, company size, requirement levels and supervisor function – a cross-sectional study in Germany | Does not meet inclusion criteria | 2115 | Kohl 2023 |
| 190 | The impact of workplace safety and customer misbehavior on supermarket workers' stress and psychological distress during the COVID-19 pandemic | Does not meet inclusion criteria | 2445 | Mayer 2022 |
| 191 | Supply chain agility responding to unprecedented changes: empirical evidence from the UK food supply chain during COVID-19 crisis | Does not meet inclusion criteria | 2480 | Quynh Nhu Do 2021 |
| 192 | Wellbeing: An HR Pathway to Sustainability * | Does not meet inclusion criteria | 2504 | Corbetta 2022 |
| 193 | PRODUCTIVITY GAINS AT SOUTHWEST TUBE. | Does not meet inclusion criteria | 195 | N/A |
| 194 | SHOCK LEADERSHIP DEVELOPMENT FOR THE MODERN ERA OF PANDEMIC MANAGEMENT AND PREPAREDNESS | Does not meet inclusion criteria | 2689 | Shufutinsky 2020 |
| 195 | Investigating associations between physical activity and presenteeism: a scoping review protocol. | Does not meet inclusion criteria | 25 | Hervieux 2020 |
| 196 | Telecommuting Comes of Age. | Does not meet inclusion criteria | 175 | Gragg 2004 |
| 197 | attendance. | Does not meet inclusion criteria | 209 | N/A |
| 198 | The perceived impact of a global pandemic on a provincial department’s organisational structure | Does not meet inclusion criteria | 2393 | Nyanhongo 2023 |
| 199 | Tildrakizumab improves high burden skin symptoms, impaired sleep and quality of life of moderate-to-severe plaque psoriasis patients in conditions close to clinical practice | Does not meet inclusion criteria | 2146 | Costanzo 2023 |
| 200 | Effect of cognitive behavioral therapy for insomnia in workers: A systematic review and meta-analysis of randomized controlled trials | Does not meet inclusion criteria | 2152 | Takano 2023 |
| 201 | Human strengths: a systematic conceptual review, conceptualizing employee strengths at work and a framework for management development | Does not meet inclusion criteria | 2722 | Vikas Rai Bhatnagar 2021 |
| 202 | Evaluating the Criteria Used for Identification of PMS. | Does not meet inclusion criteria | 95 | Dean 2006 |
| 203 | Brief report: Intellectual and academic functioning in pediatric chronic kidney disease. | Does not meet inclusion criteria | 96 | Duquette 2007 |
| 204 | The relationship of selected academic and educational factors on student attendance. | Does not meet inclusion criteria | 88 | McBride 2011 |
| 205 | Ten questions concerning the impact of environmental stress on office workers | Does not meet inclusion criteria | 2170 | Awada 2023 |
| 206 | Extracurricular activities in medical education: an integrative literature review | Does not meet inclusion criteria | 2248 | Kim 2023 |
| 207 | Malaysia Pharmaceuticals Report - Q3 2023 | Does not meet inclusion criteria | 261 | N/A |
| 208 | Testing two methods to create comparable scale scores between the Job Content Questionnaire (JCQ) and JCQ-like questionnaires in the European JACE study. | Does not meet inclusion criteria | 98 | Karasek 2007 |
| 209 | Is Smart Working Beneficial for Workers’ Wellbeing? A Longitudinal Investigation of Smart Working, Workload, and Hair Cortisol/Dehydroepiandrosterone Sulfate during the COVID-19 Pandemic | Does not meet inclusion criteria | 2111 | Falco 2023 |
| 210 | Analysis of the COVID-19 pandemic’s impacts on manufacturing: a systematic literature review and future research agenda | Does not meet inclusion criteria | 2569 | Ardolino 2022 |
| 211 | Oral health in the context of prevention of absenteeism and presenteeism in the workplace. | Does not meet inclusion criteria | 61 | Lima 2019 |
| 212 | Restaurant employees' attitudinal reactions to social distancing difficulties: a multi-wave study | Does not meet inclusion criteria | 2568 | Meng-LongHuo 2022 |
| 213 | Professional social media-enabled productivity: a five-wave longitudinal study on the role of professional social media invasion, work engagement and work exhaustion | Does not meet inclusion criteria | 2592 | Oksa 2022 |
| 214 | Presenteeism levels among Italian nurses. A multicentric survey. | Does not meet inclusion criteria | 47 | Simonetti 2021 |
| 215 | The importance of individuals' repertoires of behaviors: The scientific appropriateness of studying multiple behaviors and general attitudes. | Does not meet inclusion criteria | 107 | Hanisch 1998 |
| 216 | The relationship between commitment forms and work outcomes: A comparison of three models. | Does not meet inclusion criteria | 110 | Cohen 2000 |
| 217 | Managing employee absence. | Does not meet inclusion criteria | 111 | Walker 1997 |
| 218 | Workplace Health Promotion, Employee Wellbeing and Loyalty during Covid-19 Pandemic—Large Scale Empirical Evidence from Hungary | Does not meet inclusion criteria | 2366 | Gorgenyi-Hegyes 2021 |
| 219 | Any More Unmeeting Twains? | Does not meet inclusion criteria | 250 | Revans 1985 |
| 220 | Burden and Unmet Needs in Migraine Patients: Results from the OVERCOME (Spain) Study | Does not meet inclusion criteria | 2120 | Pascual 2023 |
| 221 | Menstruation and social inequities in Spain: a cross-sectional online survey-based study | Does not meet inclusion criteria | 2243 | Medina-Perucha 2023 |
| 222 | Compilation of References | Does not meet inclusion criteria | 2271 | N/A |
| 223 | The evolution and reconciliation of taxonomic strategies for school refusal behavior. | Does not meet inclusion criteria | 113 | Kearney 1996 |
| 224 | Posttraumatic stress in organizations: Types, antecedents, and consequences. | Does not meet inclusion criteria | 224 | Williams 2020 |
| 225 | When Quitting is Not an Option: Unpacking the Links Between Workplace Mistreatment and Withdrawal. | Does not meet inclusion criteria | 254 | McKouen 2016 |
| 226 | Teleworking benefits companies as well as individuals. | Does not meet inclusion criteria | 171 | N/A |
| 227 | Chronicling teacher's experiences in the teaching of economics in the Covid-19 era | Does not meet inclusion criteria | 2493 | Adu 2022 |
| 228 | Well-being and career change intention: COVID-19’s impact on unemployed and furloughed hospitality workers | Does not meet inclusion criteria | 2601 | Chun-Chu(Bamboo)Chen 2021 |
| 229 | The burden of pain on employee health and productivity at a major provider of business services. | Does not meet inclusion criteria | 41 | Allen 2005 |
| 230 | HEALTH CARE ADMINISTRATION DIVISION. | Does not meet inclusion criteria | 230 | N/A |
| 231 | Managing Complex Crises | Does not meet inclusion criteria | 2684 | Bratianu 2020 |
| 232 | The Impact of COVID-19 and Its Policy Responses on Local Economy and Health Conditions | Does not meet inclusion criteria | 2738 | Gungoraydinoglu 2021 |
| 233 | Stress Testing Governance | Does not meet inclusion criteria | 2667 | Van Loo 2022 |
| 234 | Doing good for organization but feeling bad: when and how narcissistic employees get prone to shame and guilt | Does not meet inclusion criteria | 2412 | Khawaja 2023 |
| 235 | The Future of Work Is Through Workforce Ecosystems | Does not meet inclusion criteria | 2716 | Altman 2021 |
| 236 | ASSESSING QUALITY OF WORK LIFE TOWARD JUNIOR HIGH SCHOOL TEACHER DURING PANDEMIC COVID-19 | Does not meet inclusion criteria | 2752 | Fakhri 2021 |
| 237 | Profile and quality of life of the adult population in good health according to the level of vitality: European NHWS cross sectional analysis. | Does not meet inclusion criteria | 22 | Tardy 2023 |
| 238 | Faker's Dozen. | Does not meet inclusion criteria | 190 | N/A |
| 239 | Bibliography | Does not meet inclusion criteria | 268 | N/A |
| 240 | Identification of internal dynamics of Türkiye’s furniture industry in the context of lean manufacturing integration maturity | Does not meet inclusion criteria | 2562 | Angin 2022 |
| 241 | The Norwegian version of the Copenhagen Psychosocial Questionnaire (COPSOQ III): Initial validation study using a national sample of registered nurses | Does not meet inclusion criteria | 2144 | Ose 2023 |
| 242 | ‘Making every contact count’ with patients with musculoskeletal conditions: a qualitative exploration of acceptability to physiotherapists | Does not meet inclusion criteria | 2210 | Parchment 2023 |
| 243 | Evaluating a multimodal, clinical and work-directed intervention (RTW-PIA) to support sustainable return to work among employees with mental disorders: study protocol of a multicentre, randomised controlled trial | Does not meet inclusion criteria | 2227 | Starke 2023 |
| 244 | Who do you trust? | Does not meet inclusion criteria | 179 | Johnson 2014 |
| 245 | Work Redesign for the 21st Century: Promising Strategies for Enhancing Worker Well-Being | Does not meet inclusion criteria | 2616 | Lovejoy 2021 |
| 246 | Maintaining a healthy workplace. | Does not meet inclusion criteria | 126 | Downey 2012 |
| 247 | Understanding drivers of influenza-like illness presenteeism within training programs: A survey of trainees and their program directors. | Does not meet inclusion criteria | 56 | Cowman 2019 |
| 248 | HOW COGENT CONDITIONS COULD FAVOR OR DISCOURAGE AGILITY ON THE JOB: A PSEUDO SMART-WORKING-SATISFACTION | Does not meet inclusion criteria | 2691 | Rainero 2021 |
| 249 | The impact of climate change on budget balances and debt in the Middle East and North Africa (MENA) region | Does not meet inclusion criteria | 2631 | Giovanis 2022 |
| 250 | Tools and Methodologies for the Analysis of Home-to-Work Shuttle Service Impacts: The ENEA “Casaccia” Case Study | Does not meet inclusion criteria | 2455 | Carapellucci 2023 |
| 251 | The Growing Burden of Major Depressive Disorders (MDD): Implications for Researchers and Policy Makers | Does not meet inclusion criteria | 2370 | Proudman 2021 |
| 252 | Predicting employee telecommuting preferences and job outcomes amid COVID-19 pandemic: a latent profile analysis | Does not meet inclusion criteria | 2289 | Bakaç 2023 |
| 253 | Changes in lifestyle-related behaviour during the COVID-19 pandemic in Japan: a questionnaire survey for examinees who underwent an annual health check-up | Does not meet inclusion criteria | 2191 | Kishimoto 2023 |
| 254 | Pain, Conflicted Feelings About Work, and Sickness Absence: A Prospective Study of the Effects of Number of Pain Sites and Role Conflicts on Medically Certified Sickness Absence | Does not meet inclusion criteria | 2295 | Christensen 2023 |
| 255 | AN INVESTIGATION INTO EMPLOYEE ENGAGEMENT IN THE PRIVATE SECTOR IN CYPRUS | Does not meet inclusion criteria | 2720 | Gleni 2021 |
| 256 | Searching for meaning in a disruptive world – Constructing a lexicon of the meanings of meaning | Does not meet inclusion criteria | 2398 | de Klerk 2023 |
| 257 | Building Resilience in the Face of Crisis: Lessons Learned from a Community Behavioral Healthcare Organization | Does not meet inclusion criteria | 2401 | Brenner 2022 |
| 258 | Temporary staff performance in universities: How can the employeeorganization relationship be enhanced in an institution | Does not meet inclusion criteria | 2527 | Rakotoarizaka 2022 |
| 259 | US Sick Leave In Global Context: US Eligibility Rules Widen Inequalities Despite Readily Available Solutions | Does not meet inclusion criteria | 2330 | Heymann 2021 |
| 260 | Health Management of Workers with Presenteeism is a New Challenge in Occupational Health | Does not meet inclusion criteria | 28 | Fujino 2018 |
| 261 | The Great Realization | Does not meet inclusion criteria | 2385 | Lindborg 2021 |
| 262 | Voluntary part-time and mandatory full-time telecommuting: a comparative longitudinal analysis of the impact of managerial, work and individual characteristics on job performance | Does not meet inclusion criteria | 2545 | Jamal 2022 |
| 263 | Lessons Learnt from Online Teaching and Beyond: What now? Three Countries Speak | Does not meet inclusion criteria | 2468 | Adriana Aletta Steyn 2023 |
| 264 | An Empirical Investigation of the Mediating Role of Job Involvement on Job Satisfaction and Employee Optimism Relationship | Does not meet inclusion criteria | 2404 | Rizvi 2023 |
| 265 | Prevalence, content and significance of advance care planning in nursing home patients. | Does not meet inclusion criteria | 117 | Kastbom 2022 |
| 266 | Life and Family Travel in the Time of COVID-19: Pandemic in England 2020 | Does not meet inclusion criteria | 2573 | Seaton 2022 |
| 267 | The Relationship between Workaholism and Personal Burnout in Dual-Earner Couples: An Analysis Using the Actor-Partner Interdependence Model | Does not meet inclusion criteria | 2104 | Russo 2023 |
| 268 | Physical symptoms and brain morphology: a population neuroimaging study in 12,286 pre-adolescents | Does not meet inclusion criteria | 2251 | Estévez-López 2023 |
| 269 | A Safety & Health Leader's Guide to DIVERSITY, EQUITY, INCLUSION & BELONGING | Does not meet inclusion criteria | 2344 | Carrillo 2023 |
| 270 | Hill's flock of lame ducks: Legislative wild cards. | Does not meet inclusion criteria | 255 | Hook 1992 |
| 271 | Accounting for the unaccountable – coping with COVID | Does not meet inclusion criteria | 2761 | Salterio 2020 |
| 272 | Innovative Business Strategies in the Face of COVID-19: An Approach to Open Innovation of SMEs in the Sonora Region of Mexico | Does not meet inclusion criteria | 2546 | Valdez-Juárez 2022 |
| 273 | The Prevalence and National Burden of Treatment-Resistant Depression and Major Depressive Disorder in the United States. | Does not meet inclusion criteria | 23 | Zhdanava 2021 |
| 274 | The Mental Health Implications of Corporate Social Responsibility: The Significance of the Sense-Making Process and Prosocial Motivation | Does not meet inclusion criteria | 2129 | Kim 2023 |
| 275 | CULTURE FINANCING IN THE EUROPEAN UNION IN COVID-19 TIMES | Does not meet inclusion criteria | 2747 | Nicolescu 2021 |
| 276 | Duty to treat and perceived risk of contagion during the COVID-19 pandemic: Norwegian physicians’ perspectives and experiences—a questionnaire survey | Does not meet inclusion criteria | 2507 | Karin Isaksson Rø 2022 |
| 277 | Shift Work and Quality of Life on Tourism, Hospitality and Restaurant Industry: The Portuguese Context | Does not meet inclusion criteria | 2682 | Silva 2022 |
| 278 | ‘Pawing’ uncertainty! how dogs attenuate the impact of daily hassles at work on uncertainty | Does not meet inclusion criteria | 2300 | Junça-Silva 2023 |
| 279 | Telework in Romania. Current State and Sustainable Socio-Economic Effects of Its Development | Does not meet inclusion criteria | 2322 | Marta-Christina Suciu 2022 |
| 280 | Violence against children during the COVID-19 pandemic | Does not meet inclusion criteria | 2585 | Bhatia 2021 |
| 281 | Liderazgo adaptativo y desempeño. Estrategia bajo un contexto COVID19 en una organización del sector eléctrico colombiano | Does not meet inclusion criteria | 2383 | Carlos Santiago Torner 2021 |
| 282 | Retaining and supporting employees with mental illness through inclusive organizations: lessons from five Canadian case studies | Does not meet inclusion criteria | 2360 | Gewurtz 2022 |
| 283 | Maternity protection entitlements for non-standard workers in low-and-middle-income countries and potential implications for breastfeeding practices: a scoping review of research since 2000 | Does not meet inclusion criteria | 2230 | Pereira-Kotze 2023 |
| 284 | A wearable technology solution and research agenda for housekeeper safety and health | Does not meet inclusion criteria | 2717 | Mejia 2021 |
| 285 | The right way to handle desertion. | Does not meet inclusion criteria | 200 | Bester 2019 |
| 286 | Handbook of research on cybersecurity risk in contemporary business systems | Does not meet inclusion criteria | 2264 | Adedoyin 2023 |
| 287 | YOUR EMPLOYEE PROBLEMS SOLVED! (cover story) | Does not meet inclusion criteria | 167 | N/A |
| 288 | Any time, any place? | Does not meet inclusion criteria | 170 | N/A |
| 289 | Survey: Telework options still growing. | Does not meet inclusion criteria | 137 | Lejeune 2013 |
| 290 | COVID-19 and the financial well-being and personal finances of South African households | Does not meet inclusion criteria | 2424 | Fouché 2023 |
| 291 | Employer-Provided Health Coverage Is Delivering Real Value for Consumers During the Pandemic. | Does not meet inclusion criteria | 152 | N/A |
| 292 | Risk factors for school-based presenteeism in children: a systematic review. | Does not meet inclusion criteria | 44 | Woodland 2023 |
| 293 | U.S. Naval and Marine Corps Occupations, Posttraumatic Stress Disorder, Depression Risk, and Absenteeism. | Does not meet inclusion criteria | 214 | Wells 2014 |
| 294 | RISK ANALYSIS OF THE CONSTRUCTION VENTURE IN THE ECONOMIC ASPECT. | Does not meet inclusion criteria | 229 | Tokarski 2021 |
| 295 | Predicting long-term absenteeism from work in construction industry: a longitudinal study. | Does not meet inclusion criteria | 219 | Hoonakker 2012 |
| 296 | Understanding Collaboration Requirements for Modular Construction and Their Cascading Failure Impact on Project Performance | Does not meet inclusion criteria | 2252 | AbdulNabi 2023 |
| 297 | NEW PRODUCTS/PUBLICATIONS. | Does not meet inclusion criteria | 256 | N/A |
| 298 | Economic burden of breast cancer: a case of Southern Iran | Does not meet inclusion criteria | 2208 | Jalali 2023 |
| 299 | HUMAN CAPITAL | Does not meet inclusion criteria | 2733 | Wustemann 2021 |
| 300 | THE INFLUENCE OF ISLAMIC WORK MOTIVATION, LEADERSHIP STYLE AND COMPETENCE ON JOB SATISFACTION AND EMPLOYEE PERFORMANCEGAMMARA MAKASSAR HOTELS | Does not meet inclusion criteria | 2472 | Basir 2023 |
| 301 | A rapid review of evaluated interventions to inform the development of a resource to support the resilience of care home nurses | Does not meet inclusion criteria | 2237 | Mallon 2023 |
| 302 | Psychosocial Safety and Health Hazards and Their Impacts on Offshore Oil and Gas Workers | Does not meet inclusion criteria | 2334 | Emma D’Antoine 2023 |
| 303 | Do You Feel Safe Here? The Role of Psychological Safety in the Relationship between Transformational Leadership and Turnover Intention Amid COVID-19 Pandemic | Does not meet inclusion criteria | 2508 | Abu Elnasr E Sobaih 2022 |
| 304 | Research on the predictability of crisis management activities in organizational loyalty | Does not meet inclusion criteria | 2564 | Pekkan 2022 |
| 305 | Policy Opportunities To Improve Prevention, Diagnosis, And Treatment Of Perinatal Mental Health Conditions | Does not meet inclusion criteria | 2610 | Moore 2021 |
| 306 | Review of Misbehavior in organizations: Theory, research, and management. | Does not meet inclusion criteria | 103 | Harrison 2004 |
| 307 | Higher COVID-19 Vaccination And Narrower Disparities In US Cities With Paid Sick Leave Compared To Those Without | Does not meet inclusion criteria | 2365 | Schnake-Mahl 2022 |
| 308 | The Covid-pandemic has ended. Again. | Does not meet inclusion criteria | 2454 | Oskam 2023 |
| 309 | Introduction to the Special Issue of PharmacoEconomics on Major Depressive Disorders | Does not meet inclusion criteria | 2349 | Greenberg 2021 |
| 310 | Exploring Hong Kong nurses' decision-making processes around presenteeism. | Does not meet inclusion criteria | 49 | Andres 2021 |
| 311 | Improving employee engagement through organizational culture in the travel industry: Perspective from a developing country during Covid-19 pandemic | Does not meet inclusion criteria | 2481 | Bui 2023 |
| 312 | Olive Garden's Expansion Of Paid Sick Leave During COVID-19 Reduced The Share Of Employees Working While Sick | Does not meet inclusion criteria | 2316 | Schneider 2021 |
| 313 | Is telemedicine a holy grail in healthcare policy: clinicians’ and patients’ perspectives from an Apex Institution in Western India | Does not meet inclusion criteria | 2437 | Gupta 2023 |
| 314 | Telecommuting Is a Big Hook for Potential Recruits. | Does not meet inclusion criteria | 148 | N/A |
| 315 | Telecommuting: A Two-Way Street. | Does not meet inclusion criteria | 161 | N/A |
| 316 | The Effect of Psychosocial Safety Climate on Engagement and Psychological Distress: A Multilevel Study on the Healthcare Sector | Does not meet inclusion criteria | 2582 | Platania 2022 |
| 317 | Improving employee outcomes in the remote working context: a time-lagged study on digital-oriented training, work-to-family conflict and empowering leadership | Does not meet inclusion criteria | 2187 | Pham 2023 |
| 318 | Digital technologies and circular economy practices: vital enablers to support sustainable and resilient supply chain management in the post-COVID-19 era | Does not meet inclusion criteria | 2416 | Cherrafi 2022 |
| 319 | Supply chain transparency for sustainability – an intervention-based research approach | Does not meet inclusion criteria | 2584 | Balakrishnan 2022 |
| 320 | THE EFFECT OF REDUCED PERCEIVED FINANCIAL WELL-BEING ON DOMESTIC PRODUCT PURCHASING BEHAVIOR: THE CASE OF TURKEY DURING COVID-19 | Does not meet inclusion criteria | 2696 | Paylan 2021 |
| 321 | The mediating effect of job satisfaction and knowledge sharing behaviour on job performance | Does not meet inclusion criteria | 2475 | Sasmita 2023 |
| 322 | Vulnerability and resilience in health crises. Evidence from European countries | Does not meet inclusion criteria | 2688 | Dimian 2021 |
| 323 | Learning loss and learning recovery | Does not meet inclusion criteria | 2540 | Patrinos 2022 |
| 324 | Improving Health and Reducing Absence Days at Work: Effects of a Mindfulness- and Skill-Based Leadership Intervention on Supervisor and Employee Sick Days | Does not meet inclusion criteria | 2148 | Vonderlin 2023 |
| 325 | Mitigating Moral Distress in Leaders of Healthcare Organizations: A Scoping Review | Does not meet inclusion criteria | 2339 | Hertelendy 2022 |
| 326 | The interaction effect of psychological capital on the relationship between self-driven personality and happiness at work in the present and post-COVID-19 era | Does not meet inclusion criteria | 2548 | Kawalya 2022 |
| 327 | Cold working environments a Covid-19 risk factor, report finds | Does not meet inclusion criteria | 2374 | Webber 2021 |
| 328 | Link between social distancing, cognitive dissonance, and social networking site usage intensity: a country-level study during the COVID-19 outbreak | Does not meet inclusion criteria | 2668 | Chakraborty 2021 |
| 329 | WHAT IS A HEALTHY WORKPLACE? FM’s role in people’s well-being. | Does not meet inclusion criteria | 210 | Guitea 2023 |
| 330 | The lost productivity cost of absenteeism due to COVID-19 in health care workers in Iran: a case study in the hospitals of Mashhad University of Medical Sciences | Does not meet inclusion criteria | 2448 | Faramarzi 2021 |
| 331 | THE PANDEMIC IN PRISON: IMPLICATIONS FOR CALIFORNIA POLITICS AND POLICYMAKING | Does not meet inclusion criteria | 2647 | Lerman 2020 |
| 332 | CREM perspective on home office—a consideration of the workplace and its mechanisms of action | Does not meet inclusion criteria | 2544 | Voll 2022 |
| 333 | Absencja chorobowa pracowników a zarządzanie efektywnością pracy w erze Przemysłu 4.0. | Does not meet inclusion criteria | 206 | Striker 2021 |
| 334 | Harmonizing Home and School: Exploring the Path to Work-Life Balance and Job Satisfaction among Married Teachers | Does not meet inclusion criteria | 2419 | Qasmani 2023 |
| 335 | Dysfunctional leadership: investigating employee experiences with dysfunctional leaders | Does not meet inclusion criteria | 2533 | Abalkhail 2022 |
| 336 | Organizational commitment and associated factors among health professionals working in public hospitals of southwestern Oromia, Ethiopia | Does not meet inclusion criteria | 2249 | Fantahun 2023 |
| 337 | Perceived challenges: Unfounded reasons for not forging ahead with digital human resource management practices | Does not meet inclusion criteria | 2470 | Chapano 2023 |
| 338 | The impact of COVID‐19 on hospital‐based workers influenza vaccination uptake: A two‐year retrospective cohort study | Does not meet inclusion criteria | 2511 | Albanesi 2022 |
| 339 | The links between absenteeism and commitment during downsizing. | Does not meet inclusion criteria | 188 | Savery 1998 |
| 340 | AbbVie Inc (ABBV) | Does not meet inclusion criteria | 270 | N/A |
| 341 | PANDEMI SONRASI OLUSAN VUCA ORTAMININ ÇALISAN INSAN KAYNAKLARI KAYGI DÜZEYI VE VERIMLILIGI ÜZERINE ETKISI | Does not meet inclusion criteria | 2369 | Akdemir 2021 |
| 342 | Sustained effectiveness and safety of subcutaneous tocilizumab over two years in the ARATA observational study | Does not meet inclusion criteria | 2114 | Behrens 2023 |
| 343 | Excess hospitalizations and mortality associated with seasonal influenza in Spain, 2008–2018 | Does not meet inclusion criteria | 2229 | Pumarola 2023 |
| 344 | The impact of emotional intelligence on behavioural factors during transition: A case of the Free State Technical and Vocational Education and Training colleges | Does not meet inclusion criteria | 2728 | Motlhanke 2021 |
| 345 | The Use of Decision Trees for Analysis of the Potential Determinants for the Incidence of Deaths and Cases of Coronavirus (Covid-19) in Different Countries | Does not meet inclusion criteria | 2336 | Wyrobek 2020 |
| 346 | Population-level prevalence, effect on quality of life, and treatment behavior for erectile dysfunction and premature ejaculation in Poland | Does not meet inclusion criteria | 2240 | Przydacz 2023 |
| 347 | Modelos híbridos: no es cuánto y dónde, sino quién y cómo. | Does not meet inclusion criteria | 128 | García 2021 |
| 348 | Enhancing Employee Resilience | Does not meet inclusion criteria | 2386 | Cooper 2022 |
| 349 | Sending them home to work: Telecommuting. | Does not meet inclusion criteria | 164 | Cote-O'Hara 1993 |
| 350 | Workplace rehabilitation. | Does not meet inclusion criteria | 215 | Goldman 2004 |
| 351 | Cognitive dysfunction in patients with childhood-onset systemic lupus erythematosus may impact treatment | Does not meet inclusion criteria | 2238 | TeixeiraSantos 2023 |
| 352 | Skipping school: An exploratory study of truancy. | Does not meet inclusion criteria | 106 | Wiehe 2000 |
| 353 | Causes and mitigation of academic dishonesty among healthcare students in a Nigerian university | Does not meet inclusion criteria | 2212 | Orok 2023 |
| 354 | A randomised controlled trial evaluating the Guide Cymru mental health literacy intervention programme in year 9 (age 13–14) school pupils in Wales | Does not meet inclusion criteria | 2235 | Simkiss 2023 |
| 355 | DYNAMICS OF SPA TOURISM STATISTICS IN SELECTED COUNTRIES OF CENTRAL AND EASTERN EUROPE | Does not meet inclusion criteria | 2485 | Čeperković 2023 |
| 356 | A Critical Review of Employee Health, Wellbeing Programs, and Organizational Success: Navigating Post-COVID-19 Sustainable Development | Does not meet inclusion criteria | 2392 | Vyas-Doorgapersad 2023 |
| 357 | Alternative Work Schedule Has to Be Reasonable. | Does not meet inclusion criteria | 149 | Lloyd 2012 |
| 358 | Disruptions and resilience in global container shipping and ports: the COVID-19 pandemic versus the 2008–2009 financial crisis | Does not meet inclusion criteria | 2659 | Notteboom Theo 2021 |
| 359 | Financial professionals and financial well-being: Evidence from the national financial well-being survey | Does not meet inclusion criteria | 2651 | Stebbins 2022 |
| 360 | Is more really better? Performance measure variety and environmental uncertainty | Does not meet inclusion criteria | 2737 | Rikhardsson 2021 |
| 361 | Covid-19 Impact and Population Density | Does not meet inclusion criteria | 2674 | Wang 2021 |
| 362 | Pandemic-Induced Telework Challenges and Strategies | Does not meet inclusion criteria | 5 | Greer 2023 |
| 363 | Methodological Issues in the Use of Absence Data. | Does not meet inclusion criteria | 192 | Hammer 1981 |
| 364 | Analyzing The Impact Of Job Stress, Job Insecurity, and Work Engagement On Job Performance During The COVID-19 Pandemic In The Aviation Industry | Does not meet inclusion criteria | 2541 | Paramita 2022 |
| 365 | Impact of covid-19 on the Quality of Life of University Professors of Sonora and Sinaloa (Mexico) | Does not meet inclusion criteria | 2192 | Campas 2023 |
| 366 | Bibliometric visualisation of industrial and organisational psychology during COVID-19 pandemic: Insight for future research | Does not meet inclusion criteria | 2364 | Fatuhrahmah 2022 |
| 367 | The Covid-19 outbreak, a Failure of Social Protection System: A Policy Perspective of Energy and Economic Recovery | Does not meet inclusion criteria | 2605 | Nosheen 2021 |
| 368 | Facilitation activities for change response: a qualitative study on infection prevention and control professionals during a pandemic in Brazil | Does not meet inclusion criteria | 2686 | Irgang 2021 |
| 369 | Mind the Gap: Yet More Evidence for the Importance of Education for Children With Uncontrolled Asthma | Does not meet inclusion criteria | 2611 | Homaira 2021 |
| 370 | Reflections on the impact of “flatten the curve” on interdependent workforce sectors | Does not meet inclusion criteria | 2723 | Santos 2020 |
| 371 | The Job Leeway Scale: Initial Evaluation of a Self-report Measure of Health-Related Flexibility and Latitude at Work | Does not meet inclusion criteria | 2158 | Shaw 2023 |
| 372 | The indirect costs of multiple sclerosis: systematic review and meta-analysis. | Does not meet inclusion criteria | 122 | Stawowczyk 2015 |
| 373 | Annual costs of bipolar disorders in Germany. | Does not meet inclusion criteria | 104 | Runge 2004 |
| 374 | IMPACT OF LEADERSHIP STYLES TOWARD EMPLOYEE ENGAGEMENT AMONG MALAYSIAN CIVIL DEFENCE FORCE | Does not meet inclusion criteria | 2764 | Ismail 2021 |
| 375 | The Role of Law in Addressing Poverty and Inequality in High Income Countries: A Comparative View of Menstrual Hygiene Management and Its Impact on Education and Health in the UK and Select High Income Sub-Saharan African Countries | Does not meet inclusion criteria | 2630 | Bakibinga 2021 |
| 376 | Universal Basic Income in the Spanish construction sector: Engaging businesses in a public-policy debate | Does not meet inclusion criteria | 2715 | Gómez-Frías 2021 |
| 377 | Malaysia Pharmaceuticals Report - Q2 2023 | Does not meet inclusion criteria | 262 | N/A |
| 378 | THE DETERMINATION OF PRODUCTS, PRICES, PROMOTION OF EDUCATIONAL QUALITY THROUGH THE LECTURERS SATISFACTION OF BATAM UNIVERSITY IN THE COVID 19 PANDEMIC ERA | Does not meet inclusion criteria | 2409 | Indrawan 2022 |
| 379 | Precariousness and discontinuous work history in association with health. | Does not meet inclusion criteria | 87 | Sirvio 2012 |
| 380 | Companies Devise Strategies for Flu Interruptions. | Does not meet inclusion criteria | 178 | Mattioli 2009 |
| 381 | RESEARCH NEWS ROUND-UP | Does not meet inclusion criteria | 2377 | N/A |
| 382 | Impact of COVID-19 Pandemic on Generation Z Employees’ Perception and Behavioral Intention toward Advanced Information Technologies in Hotels | Does not meet inclusion criteria | 2530 | Zhang 2022 |
| 383 | The challenge of change fatigue on workplace mental health | Does not meet inclusion criteria | 2317 | Bruce 2020 |
| 384 | Absent correctional staff: A discussion of the issue and recommendations for future research. | Does not meet inclusion criteria | 108 | Lambert 2001 |
| 385 | The Impact of COVID-19 Outbreak on Human Resource Operation: Empirical Evidence from the Perspective of Malaysian Retail Employees in Klang Valley | Does not meet inclusion criteria | 2586 | Hossin 2020 |
| 386 | Paid Sick Leave's Payoff | Does not meet inclusion criteria | 273 | Shinall 2022 |
| 387 | Does physical activity impact on presenteeism and other indicators of workplace well-being?. | Does not meet inclusion criteria | 37 | Brown 2011 |
| 388 | A multilevel perspective on the perceived effects of COVID-19 on nurses in China | Does not meet inclusion criteria | 2492 | Sun 2022 |
| 389 | ALIGNING EMPLOYEE ASSISTANCE PROGRAMME (EAP) IN THE ERA OF NEW NORMAL REALITY- AN ORGANIZATIONAL PERSPECTIVE | Does not meet inclusion criteria | 2628 | Nisha 2021 |
| 390 | Factors Associated With the Presenteeism of Single-Person Household Employees in Korea: The 5th Korean Working Conditions Survey (KWCS). | Does not meet inclusion criteria | 45 | Min 2021 |
| 391 | Instructional Time Loss in Developing Countries: Concepts, Measurement, and Implications. | Does not meet inclusion criteria | 248 | Abadzi 2009 |
| 392 | A systematic review of studies identifying predictors of poor return to work outcomes following workplace injury. | Does not meet inclusion criteria | 235 | Street 2015 |
| 393 | Extent and predictors of presenteeism among healthcare professionals working in Swiss hospitals, nursing homes and home care organizations | Does not meet inclusion criteria | 2125 | Peter 2023 |
| 394 | Artificial Intelligence and Reduced SMEs’ Business Risks. A Dynamic Capabilities Analysis During the COVID-19 Pandemic | Does not meet inclusion criteria | 2519 | Drydakis 2022 |
| 395 | NEW EVIDENCE ON THE DETERMINANTS OF ABSENTEEISM USING LINKED EMPLOYER-EMPLOYEE DATA. | Does not meet inclusion criteria | 142 | DIONNE 2007 |
| 396 | The physical office work environment and employee wellbeing: Current state of research and future research agenda | Does not meet inclusion criteria | 2302 | Kazlauskaitė 2023 |
| 397 | Identifying consumer resistance of mobile payment during COVID-19: an interpretive structural modeling (ISM) approach | Does not meet inclusion criteria | 2528 | Singh 2022 |
| 398 | Protecting vulnerable people during pandemics through home delivery of essential supplies: a distribution logistics model | Does not meet inclusion criteria | 2711 | Breitbarth 2021 |
| 399 | Occupational Health Inequalities by Issues on Gender and Social Class in Labor Market: Absenteeism and Presenteeism Across 26 OECD Countries. | Does not meet inclusion criteria | 51 | Kwon 2020 |
| 400 | COVID-19: impact on quality of work life in real estate sector | Does not meet inclusion criteria | 2474 | MajumderSoumi 2022 |
| 401 | Workplace violence among healthcare workers during COVID-19 pandemic in a Jordanian governmental hospital: the tip of the iceberg | Does not meet inclusion criteria | 2525 | Ghareeb 2021 |
| 402 | Occupational demands, environmental resources, and personal resources effects on presenteeism and health. | Does not meet inclusion criteria | 74 | Tetrick 2014 |
| 403 | Managers oppose the spread of teleworking. | Does not meet inclusion criteria | 173 | N/A |
| 404 | Design and validation of the presenteeism scale in nursing | Does not meet inclusion criteria | 2153 | Mohammadi 2023 |
| 405 | Prediction of sickness absence in patients with chronic low back pain: A systematic review | Does not meet inclusion criteria | 100 | Kuijer 2006 |
| 406 | The organizational benefits of assisting domestically abused employess | Does not meet inclusion criteria | 240 | Johnson 1999 |
| 407 | Sick leave guidance aims to bring absentees back | Does not meet inclusion criteria | 246 | N/A |
| 408 | Mental health of buisness academics within the COVID-19 era: can meaningful work help? A qualitative study | Does not meet inclusion criteria | 2532 | Mousa 2022 |
| 409 | Left to their own devices? Antecedents and contingent effects of workplace anxiety in the WFH selling environment | Does not meet inclusion criteria | 2614 | Rangarajan 2022 |
| 410 | What do hotel managers think of employee rewards? An exploration of five-star hotels in cape town | Does not meet inclusion criteria | 2484 | Cwibi 2023 |
| 411 | Benefits of a basic income for employees experiencing a mental health condition | Does not meet inclusion criteria | 2312 | Carpini 2021 |
| 412 | Integrating management control systems, mindfulness and sustainability: an occupational health and safety perspective | Does not meet inclusion criteria | 2704 | Wahab Shahbaz 2021 |
| 413 | Experiences of referral with an obstetric emergency: Voices of women admitted at Mbarara Regional Hospital, South Western Uganda | Does not meet inclusion criteria | 2206 | Nabulo 2023 |
| 414 | Learning complex technology online: Effect of challenge and hindrance techno-stressors on student satisfaction and retention | Does not meet inclusion criteria | 2452 | Yu (Audrey) Zhao 2023 |
| 415 | Troubling gender norms on Mumsnet: Working from home and parenting during the UK's first COVID lockdown | Does not meet inclusion criteria | 2166 | Handley 2023 |
| 416 | The socio-economic management control after the COVID-19 pandemic | Does not meet inclusion criteria | 2542 | Nassimi 2022 |
| 417 | Universal symptom monitoring to address presenteeism in healthcare workers | Does not meet inclusion criteria | 48 | Lichtman 2021 |
| 418 | A social policy case for a four-day week | Does not meet inclusion criteria | 2467 | Chung 2022 |
| 419 | Burnout, engagement, y la perception sobre practicas de gestion en pandemica por COVID-19 que tienes trabajadores del centro sur de Chile | Does not meet inclusion criteria | 2512 | Acuña-Hormazabal 2021 |
| 420 | School-based restorative justice: Lessons and opportunities in a post-pandemic world | Does not meet inclusion criteria | 2662 | Velez 2021 |
| 421 | Managing the mind, body, and soul-closing the gap between managers and young employees with anxiety disorders | Does not meet inclusion criteria | 2731 | Hunter 2021 |
| 422 | School refusal behavior: The examination of a Las Vegas truancy diversion program | Does not meet inclusion criteria | 86 | Chapman 2012 |
| 423 | Requested to do right things excessively: how citizenship pressure/future focus influence health-related work outcomes in health organizations during the pandemic | Does not meet inclusion criteria | 2476 | Junbang Lan 2022 |
| 424 | The Gender Life Satisfaction/Depression Paradox | Does not meet inclusion criteria | 2594 | Becchetti Leonardo 2022 |
| 425 | Recovery Experiences for Work and Health Outcomes: A Meta-Analysis and Recovery-Engagement-Exhaustion Model | Does not meet inclusion criteria | 2157 | Headrick 2023 |
| 426 | Job Insecurity, Job Instability, and Job Satisfaction in the Context of the COVID-19 Pandemic | Does not meet inclusion criteria | 2700 | Marcela-SeforaNemteanu 2021 |
| 427 | Opening the Workplace After COVID-19: What Lessons Can be Learned from Return-to-Work Research? | Does not meet inclusion criteria | 2354 | Shaw 2020 |
| 428 | Development and Validation of a Questionnaire to Assess Self-Endangering Work Behavior | Does not meet inclusion criteria | 2150 | Mustafić 2023 |
| 429 | The dualistic view of challenge-hindrance technostress in accounting information systems: Technological antecedents and coping responses | Does not meet inclusion criteria | 2189 | Zhu 2023 |
| 430 | COVID-19-related job insecurity and employees’ behavioral outcomes: mediating role of emotional exhaustion and moderating role of symmetrical internal communication | Does not meet inclusion criteria | 2417 | Abdul Karim Khan 2022 |
| 431 | The Need for Novel Approaches in Assessing the Value of COVID-19 Vaccines | Does not meet inclusion criteria | 2319 | Angelis 2021 |
| 432 | Depression in the context of workplace injury | Does not meet inclusion criteria | 75 | Ferguson 2012 |
| 433 | COVID-19 and Economics Forecasting on Advanced and Emerging Countries | Does not meet inclusion criteria | 2632 | García 2021 |
| 434 | The Response of the EU Agri-Food Chain to the COVID-19 Pandemic:Chronicles from the EU and Selected Member States | Does not meet inclusion criteria | 2742 | Montanari 2020 |
| 435 | Mental health experiences of healthcare professionals during COVID-19 | Does not meet inclusion criteria | 2645 | Cook 2021 |
| 436 | Family Leave Policies and Employee Attitudinal Outcomes in Korea: The Moderating and Joint Moderating Effects of Gender and Marital Status | Does not meet inclusion criteria | 2256 | Jeon 2023 |
| 437 | Association of strong opioids and antibiotics prescribing with GP burnout: | Does not meet inclusion criteria | 2131 | Hodkinson 2023 |
| 438 | Telecommuting in the 21st Century: Benefits, Issues, and a Leadership Model Which Will Work. | Does not meet inclusion criteria | 140 | N/A |
| 439 | The ubiquitous effects of financial stress during pandemics and beyond: Opportunities for industrial and organizational psychology | Does not meet inclusion criteria | 2745 | Phetmisy 2021 |
| 440 | Ergonomic assessment for designing manual material handling tasks at a food warehouse in India: A case study | Does not meet inclusion criteria | 2242 | Adhaye 2023 |
| 441 | The top 100 cited articles in menstrual health among adolescent girls: a citation analysis | Does not meet inclusion criteria | 2245 | Alekhya 2023 |
| 442 | On different pages? More dangerous than a papercut: Implications of incongruence between leader and member safety climate perceptions | Does not meet inclusion criteria | 2117 | Lee 2023 |
| 443 | A. (No title provided) | Does not meet inclusion criteria | 197 | Statt 1999 |
| 444 | Resilency and leadership in organizations | Does not meet inclusion criteria | 2373 | Ayoko 2021 |
| 445 | Resuming Operations During a Pandemic | Does not meet inclusion criteria | 2591 | N/A |
| 446 | WHAT WAS THE COVID-19 PANDEMIC'S IMPACT ON HUMAN RESOURCE MANAGEMENT AND WORK? AN INTEGRATIVE LITERATURE REVIEW | Does not meet inclusion criteria | 2413 | Presti 2023 |
| 447 | Lessons in absenteeism management. | Does not meet inclusion criteria | 186 | Ericson 2001 |
| 448 | Knowledge, attitudes and practices of health care workers in a cardiology department on influenza vaccination | Does not meet inclusion criteria | 2260 | Benedict Kpozehouen 2023 |
| 449 | Together alone: staying well in challenging times | Does not meet inclusion criteria | 2329 | Ramlakhan 2021 |
| 450 | Determinants of the intention to seek psychotherapeutic consultation at work - a cross-sectional study in Germany | Does not meet inclusion criteria | 2223 | Kohl 2023 |
| 451 | Teleworkers' job performance: a study examining the role of age as an important diversity component of companies' workforce | Does not meet inclusion criteria | 2272 | Hamouche 2023 |
| 452 | How job insecurity affects emotional exhaustion? A study of job insecurity rumination and psychological capital during COVID-19 | Does not meet inclusion criteria | 2741 | Konkel 2021 |
| 453 | Work from home and the association with sedentary behaviors, leisure-time and domestic physical activity in the ELSA-Brasil study | Does not meet inclusion criteria | 2179 | de Oliveira da Silva Scaranni 2023 |
| 454 | Relationship between alcohol consumption and telecommuting preference‐practice mismatch during the COVID‐19 pandemic | Does not meet inclusion criteria | 2332 | Watanabe 2022 |
| 455 | [Presenteeism]. | Does not meet inclusion criteria | 34 | Malinska 2013 |
| 456 | Prevalence of Presenteeism in Agricultural Workers: Systematic Review | Does not meet inclusion criteria | 2106 | Siqueira 2023 |
| 457 | How coronavirus (COVID-19) pandemic thought concern affects employees’ work performance: evidence from real time survey | Does not meet inclusion criteria | 2510 | Mahfoudh Hussein Mgammal 2022 |
| 458 | Double-Edged Sword Effect of Flexible Work Arrangements on Employee Innovation Performance: From the Demands–Resources–Individual Effects Perspective | Does not meet inclusion criteria | 2197 | Wang 2023 |
| 459 | Impact of a Caregiver-Friendly Workplace Policies Intervention: A Prospective Economic Evaluation. | Does not meet inclusion criteria | 55 | Mofidi 2019 |
| 460 | Efficacy of an Online Workplace Mental Health Accommodations Psychoeducational Course: A Randomized Controlled Trial | Does not meet inclusion criteria | 2186 | Faller 2023 |
| 461 | Cultural Distances and Its Association to Time Spent on Conflicts | Does not meet inclusion criteria | 266 | Dirrler 2023 |
| 462 | Promoting physical activity-related health competence to increase leisure-time physical activity and health-related quality of life in German private sector office workers | Does not meet inclusion criteria | 2250 | Blaschke 2023 |
| 463 | What is needed to sustain improvements in hospital practices post-COVID-19? a qualitative study of interprofessional dissonance in hospital infection prevention and control | Does not meet inclusion criteria | 2534 | Gilbert 2022 |
| 464 | Work–life balance and life satisfaction among the self-employed | Does not meet inclusion criteria | 2697 | Best 2021 |
| 465 | The effect of COVID-19 on foreign direct investment inflows: stylised facts and some explanations | Does not meet inclusion criteria | 2420 | Moosa 2022 |
| 466 | Neuroticism mediates the association between childhood abuse and the well-being of community dwelling adult volunteers | Does not meet inclusion criteria | 2147 | Fujimura 2023 |
| 467 | Uncovering the affective affordances of videoconference technologies | Does not meet inclusion criteria | 2589 | Vidolov 2022 |
| 468 | Tech and the times: why technology is the double-edged sword that HR teams must navigate | Does not meet inclusion criteria | 2335 | Latus 2022 |
| 469 | Coronavirus: caring for the carers | Does not meet inclusion criteria | 2375 | Hilder 2020 |
| 470 | Cost effectiveness review of text messaging, smartphone application, and website interventions targeting T2DM or hypertension | Does not meet inclusion criteria | 2239 | Willems 2023 |
| 471 | Multiplier Effects and Compensation Mechanisms for Inclusion in Health Economic Evaluation: A Systematic Review | Does not meet inclusion criteria | 2124 | Krol 2023 |
| 472 | The Role of Space and Place in Organizational and Institutional Change: A Systematic Review of the Literature | Does not meet inclusion criteria | 2201 | Wright 2023 |
| 473 | Using blockchain technology to drive operational excellence in perishable food supply chains during outbreaks | Does not meet inclusion criteria | 2523 | Kayikci 2022 |
| 474 | Work and children in Spain: challenges and opportunities for equality between men and women | Does not meet inclusion criteria | 2314 | Hupkau 2022 |
| 475 | Teacher professionalism during the pandemic: Courage, care and resilience | Does not meet inclusion criteria | 2204 | Day 2023 |
| 476 | Compassionate leadership: how to support your team when fixing the problem seems impossible | Does not meet inclusion criteria | 2350 | N/A |
| 477 | Meaningful work from ethics perspective: Examination of ethical antecedents and outcomes of meaningful work | Does not meet inclusion criteria | 2222 | Rai 2023 |
| 478 | Tackling long-term absence (2): London Borough of Brent. | Does not meet inclusion criteria | 201 | N/A |
| 479 | Should I stay or should I go? Why nurses are leaving community nursing in the UK | Does not meet inclusion criteria | 2431 | Senek 2023 |
| 480 | The FLURESP European commission project: cost-effectiveness assessment of ten public health measures against influenza in Italy: is there an interest in COVID-19 pandemic? | Does not meet inclusion criteria | 2440 | Beresniak 2023 |
| 481 | Determinants of mental health: Role of organisational climate and decent work amongst employees | Does not meet inclusion criteria | 2432 | Ruzungunde 2023 |
| 482 | Perspectives on workplace communication and well-being in hybrid work environments | Does not meet inclusion criteria | 2274 | Duarte 2023 |
| 483 | The influence of COVID-19 on employee ergonomics and employee engagement of banking employees | Does not meet inclusion criteria | 2536 | Kulkarni 2022 |
| 484 | Australia Firms Boost Ads for Four-Day Week to Lure Talent. | Does not meet inclusion criteria | 150 | Argana 2023 |
| 485 | Impact of flexible work arrangements on employees’ perceived productivity, organisational commitment and perceived work quality: a United Arab Emirates case-study | Does not meet inclusion criteria | 2269 | Hashmi 2023 |
| 486 | COVID-19 is a moderating variable with its own moderating factors | Does not meet inclusion criteria | 2313 | Kniffin 2021 |
| 487 | A hotel stay for a respite from work? Examining recovery experience, rumination and well-being among hotel and bed-and-breakfast guests | Does not meet inclusion criteria | 2557 | Chun-Chu 2022 |
| 488 | Artificial intelligence as a fundamental tool in management of infectious diseases and its current implementation in COVID-19 pandemic | Does not meet inclusion criteria | 2613 | KaurIshnoor 2021 |
| 489 | Who worked from home in Brazil? Inequalities highlighted by the pandemic | Does not meet inclusion criteria | 2680 | Nicole Rennó Castro 2021 |
| 490 | Human Sustainability and Work: A Meta-Synthesis and New Theoretical Framework | Does not meet inclusion criteria | 2151 | Barnes 2023 |
| 491 | A moderated-mediation model of individual learning and commitment: evidence from healthcare industry in India (part II) | Does not meet inclusion criteria | 2683 | Karthikeyan Somaskandan 2022 |
| 492 | Teacher absenteeism and ill health retirement: A review. | Does not meet inclusion criteria | 109 | Bowers 2001 |
| 493 | Easing the pressure. (cover story) | Does not meet inclusion criteria | 252 | Malamatenios 2013 |
| 494 | The prevalence of gastrointestinal symptoms and cobalamin deficiency in patients with chronic urticaria | Does not meet inclusion criteria | 2134 | Abadeh 2023 |
| 495 | Evaluating the validity of emergency department influenza-like illness (ILI) syndrome for influenza in children and its use in a surveillance setting. | Does not meet inclusion criteria | 85 | Rodriguez 2012 |
| 496 | Malaysia Pharmaceuticals Report - Q4 2023 | Does not meet inclusion criteria | 259 | N/A |
| 497 | Idorsia Pharmaceutical Ltd (IDIA) | Does not meet inclusion criteria | 271 | N/A |
| 498 | Female Reproductive Health and Contraception Use in CKD: An International Mixed-Methods Study | Does not meet inclusion criteria | 2130 | Rodriguez 2012 |
| 499 | Happiness at work, organization citizenship behaviour and workplace diversity: a study on Indian private sector bank employees | Does not meet inclusion criteria | 2490 | Singh 2022 |
| 500 | How Are Nonprofit Workers Doing? Exploring the Personal and Professional Impact of COVID-19 | Does not meet inclusion criteria | 2514 | Kuenzi 2022 |
| 501 | Oklahoma Redefines Employee Misconduct. | Does not meet inclusion criteria | 232 | N/A |
| 502 | Female academics' views on work-life conflict in a Covid-19 global pandemic in Nigeria | Does not meet inclusion criteria | 2666 | Adewumi 2021 |
| 503 | Keeping nurses engaged during COVID-19: An i-deal perspective | Does not meet inclusion criteria | 2441 | Ngobeni 2022 |
| 504 | Individual-Level Market Orientation of Healthcare Practitioners in the COVID-19 Era | Does not meet inclusion criteria | 2477 | Kilic 2023 |
| 505 | TASK PERFORMANCE AND JOB SATISFACTION UNDER THE EFFECT OF REMOTE WORKING: CALL CENTER EVIDENCE | Does not meet inclusion criteria | 2423 | Aslan 2022 |
| 506 | Age shall not wither them. | Does not meet inclusion criteria | 169 | N/A |
| 507 | New York City Earned Sick Time Act Expanded Before Taking Effect. | Does not meet inclusion criteria | 205 | N/A |
| 508 | Workplace flexibility, work–family interface, and psychological distress: differences by family caregiving obligations and gender | Does not meet inclusion criteria | 2200 | Yucel 2023 |
| 509 | Evaluation of the socioprofessional consequences of thoracic outlet syndrome | Does not meet inclusion criteria | 2247 | Logiou 2023 |
| 510 | First to React Is the Last to Forgive: Evidence from the Stock Market Impact of COVID 19 | Does not meet inclusion criteria | 2719 | N/A |
| 511 | Who Gains Mental Health Benefits from Work Autonomy? The Roles of Gender and Occupational Class | Does not meet inclusion criteria | 2281 | Lu 2023 |
| 512 | Leadership, management and organisational implications for public service employee well-being and performance | Does not meet inclusion criteria | 2456 | Haricharan 2023 |
| 513 | COVID-19 impact on hospitality retail employees’ turnover intentions | Does not meet inclusion criteria | 2500 | McCartney 2022 |
| 514 | WITHDRAWN: Needed: less influenza vaccine hesitancy and less presenteeism among health care workers in the COVID-19 era. | Does not meet inclusion criteria | 42 | Grech 2020 |
| 515 | Job Stress, Emotional Exhaustion, Job Satisfaction, and Turnover Intentions: The Role of Servant Leadership | Does not meet inclusion criteria | 2653 | Johnson 2022 |
| 516 | Chapter 3. Working conditions of key workers | Does not meet inclusion criteria | 267 | N/A |
| 517 | The nurse without a nurse: the antecedents of presenteeism in nursing. | Does not meet inclusion criteria | 43 | Mohammadi 2021 |
| 518 | Effective nurse leadership in times of crisis | Does not meet inclusion criteria | 2671 | N/A |
| 519 | School absenteeism and school refusal behaviour in youth: A contemporary review | Does not meet inclusion criteria | 93 | Kearney 2008 |
| 520 | Presenteeism, Absenteeism, and Lost Work Productivity among Depressive Patients from Five Cities of Colombia. | Does not meet inclusion criteria | 58 | Uribe 2017 |
| 521 | Assessing the impact of grief on quality of life, work productivity, and health outcomes for parents bereaved from SMA: A study protocol | Does not meet inclusion criteria | 264 | Riley 2023 |
| 522 | High Anxiety Warning from HEA Group. | Does not meet inclusion criteria | 245 | N/A |
| 523 | #TreatmentResistantDepression: A qualitative content analysis of Tweets about difficult-to-treat depression | Does not meet inclusion criteria | 2123 | Talbot 2023 |
| 524 | Facilitating the employment of people with mental health difficulties in Ireland | Does not meet inclusion criteria | 2718 | Tighe 2021 |
| 525 | Who Counts? Educational Disadvantage among Children Identified as Homeless and Implications for the Systems That Serve Them. | Does not meet inclusion criteria | 217 | Lowell 2022 |
| 526 | Sickness absence around contact with outpatient mental health care services - differences between migrants and non-migrants: a Norwegian register study | Does not meet inclusion criteria | 2207 | Straiton 2023 |
| 527 | Lay representations of workplace stress: What do people really mean when they say they are stressed? | Does not meet inclusion criteria | 241 | Kinman 2005 |
| 528 | Understanding and exploring the cost of poor mental health at work for organizations and society | Does not meet inclusion criteria | 2254 | Hassard 2023 |
| 529 | The route to well-being at workplace: examining the role of job insecurity and its antecedents | Does not meet inclusion criteria | 2415 | Saeed 2023 |
| 530 | Does social distancing make the heart grow fonder? The impact of work spouse interaction frequency on organizational outcomes | Does not meet inclusion criteria | 2559 | RestKlaus-Dieter 2022 |
| 531 | Enhancing Competitive Advantage in These Pandemic Times Through 5S Concept: The Case of a Rubber Company in Vietnam | Does not meet inclusion criteria | 2695 | Lan et al., 2021 |
| 532 | Mental health and mental illness in organizations: a review, comparison and extension | Does not meet inclusion criteria | 2112 | Cronin 2023 |
| 533 | The incidence and magnitude of the health costs of in-person schooling during the COVID-19 pandemic | Does not meet inclusion criteria | 2621 | Mulligan 2021 |
| 534 | An unintended consequence of flexible work arrangements (FWAs) - the role of peer resentment on turnover intentions in public accounting | Does not meet inclusion criteria | 2261 | Calhoun 2023 |
| 535 | Applying the sociotechnical systems theory to crowdsourcing food delivery platforms: the perspective of crowdsourced workers | Does not meet inclusion criteria | 2634 | Lee et al., 2022 |
| 536 | Impact of employees’ internal factors and job performance on organizational commitment in government organizations during COVID-19: Evidence from Kuwait | Does not meet inclusion criteria | 2520 | Aldasem 2022 |
| 537 | Employee Engagement in Organisations During a Pandemic | Does not meet inclusion criteria | 2702 | Kot-Radojewska & Wódz 2021 |
| 538 | Roles of trade logistics to the development of international trade: A perspective of Nigeria | Does not meet inclusion criteria | 2615 | Salawu 2022 |
| 539 | COVID-19: Considering impacts to employees and the workplace | Does not meet inclusion criteria | 2669 | Bussin & Swart-Opperman 2021 |
| 540 | The impact of corruption on victim teachers during promotion processes in South Africa's basic education system | Does not meet inclusion criteria | 2443 | Rammbuda 2023 |
| 541 | Pandemic stress and the role of resources in expatriate-local interaction adjustment: an extension of Berry's model | Does not meet inclusion criteria | 2572 | Reade & McKenna 2022 |
| 542 | The Impact of the Covid-19 Pandemic on the Application of Management Methods by Industrial Goods Processing Enterprises | Does not meet inclusion criteria | 2708 | Motała 2021 |
| 543 | The Effect of COVID-19 on the Hospitality Industry: The Implication for Open Innovation | Does not meet inclusion criteria | 2609 | Khan et al., 2021 |
| 544 | Transformational leadership influences on organisational justice and employee commitment in a customer service organisation | Does not meet inclusion criteria | 2439 | Khuzwayo 2023 |
| 545 | Consumer information technology use in the post-pandemic workplace: a post-acceptance adaptation perspective | Does not meet inclusion criteria | 2156 | Yin et al., 2023 |
| 546 | Embracing Changes: Virtual Change Management and Leadership Training Implications | Does not meet inclusion criteria | 2566 | Chen & Hix 2022 |
| 547 | The effects of on-the-job embeddedness and its sub-dimensions on small-sized hotel employees' organizational commitment, work engagement and turnover intentions | Does not meet inclusion criteria | 2663 | Ampofo et al., 2022 |
| 548 | Fragmented But Widespread Microconflicts: Current Limits and Future Possibilities for Organizing Precarious Workers in the French Logistics Sector | Does not meet inclusion criteria | 2639 | Benvegnù et al., 2022 |
| 549 | Managerial support, work-family conflict and employee outcomes: an Australian study | Does not meet inclusion criteria | 2258 | Tran et al., 2023 |
| 550 | Moral Stress: Considering the Nature and Effects of Managerial Moral Uncertainty. | Does not meet inclusion criteria | 234 | Reynolds et al., 2012 |
| 551 | The Caregiver Conundrum | Does not meet inclusion criteria | 2396 | Rehaut 2023 |
| 552 | The impact of ethical climate and the LMX relationship on nurses' burnout in hospitals in Serbia | Does not meet inclusion criteria | 2388 | Zorić et al., 2023 |
| 553 | Whether and how top management create flexibility in mental healthcare organizations: COVID-19 as a test case | Does not meet inclusion criteria | 2428 | van Gool et al., 2022 |
| 554 | Workplace Diversity Influence on Employee Retention in Organizations during the COVID-19 | Does not meet inclusion criteria | 2623 | Rodprayoon & Maj 2021 |
| 555 | Teaching from a distance: challenges in classroom management to promote professionalism | Does not meet inclusion criteria | 2347 | Harper & Robinson 2022 |
| 556 | Pandemics: Implications for research and practice in industrial and organizational psychology | Does not meet inclusion criteria | 2333 | Rudolph et al., 2021 |
| 557 | Covid-19 and Employee Surveillance | Does not meet inclusion criteria | 2698 | Blumenfeld et al., 2020 |
| 558 | Identifying the impact of employer branding in the retention of nurses: the mediating role of organizational culture and career development | Does not meet inclusion criteria | 2145 | Goyal & Kaur 2023 |
| 559 | Experiences of nurses and midwives with disabilities: A scoping review | Does not meet inclusion criteria | 2205 | Baker et al., 2023 |
| 560 | Human Resources Strategies & Lessons Learned During the COVID-19 Pandemic: A Literature Review | Does not meet inclusion criteria | 2556 | Plater et al., 2022 |
| 561 | Risk factors associated with respiratory infectious disease-related presenteeism: a rapid review. | Does not meet inclusion criteria | 46 | Daniels et al., 2021 |
| 562 | THE MODERATING EFFECT OF COVID-19 ON THE RELATIONSHIP BETWEEN WORK-LIFE BALANCE AND HOTEL EMPLOYEES' FEARS | Does not meet inclusion criteria | 2389 | Zaki 2022 |
| 563 | Pandemic outbreaks and food supply chains in developing countries: A case of COVID-19 in Zimbabwe | Does not meet inclusion criteria | 2414 | Chari et al., 2022 |
| 564 | Smart telework optionscan reduce absenteeism. | Does not meet inclusion criteria | 136 | N/A |
| 565 | Explaining variations in the findings of presenteeism research: A meta-analytic investigation into the moderating effects of construct operationalization and chronic health. | Does not meet inclusion criteria | 70 | McGregor et al., 2018 |
| 566 | The impact of COVID-19 pandemic on conventional work settings | Does not meet inclusion criteria | 2340 | Diab-Bahman & Al-Enzi, 2020 |
| 567 | Legal checklist. | Does not meet inclusion criteria | 220 | Aikin 1999 |
| 568 | Psychosocial burden in nurses working in nursing homes during the Covid-19 pandemic: a cross-sectional study with quantitative and qualitative data | Does not meet inclusion criteria | 2305 | Schulze et al., 2022 |
| 569 | Self-reported food safety knowledge and practices of early-school-aged children - a result of analysis in towns near the Warsaw city | Does not meet inclusion criteria | 2748 | Tomaszewska et al., 2021 |
| 570 | Contextualising over-engagement in work: Towards a more global understanding of workaholism as an addiction. | Does not meet inclusion criteria | 84 | Griffiths & Karanika-Murray, 2012 |
| 571 | The effect of consecutive ambient air pollution on the hospital admission from chronic obstructive pulmonary disease in the Chengdu region, China | Does not meet inclusion criteria | 2730 | Zhang et al., 2021 |
| 572 | NEGOTIATED QWL ISSUES AND THEIR IMPACT ON PRODUCTIVITY AND ABSENTEEISM: TESTING FOR THE VALENCE OF A KEY OPERATIONAL DEFINITION. | Does not meet inclusion criteria | 184 | Williamson and Alexander III, 1986 |
| 573 | Job demands and resources when using technologies at work - development of a digital work typology | Does not meet inclusion criteria | 2268 | Ruiner et al., 2023 |
| 574 | The COVID-19 pandemic: the woes of small construction firms in Ghana | Does not meet inclusion criteria | 2425 | Amoah et al., 2022 |
| 575 | Measures to strengthen primary health-care systems in low- and middle-income countries | Does not meet inclusion criteria | 2756 | Langlois et al., 2020 |
| 576 | School Refusal. | Does not meet inclusion criteria | 94 | Heyne 2006 |
| 577 | ON THE CAUSAL ORDERING OF JOB SATISFACTION AND ORGANIZATIONAL COMMITMENT. | Does not meet inclusion criteria | 227 | Curry et al., 1986 |
| 578 | A Checklist to Curb Attendance Problems | Does not meet inclusion criteria | 257 | N/A |
| 579 | School Board Not Liable for Disciplining Disabled Educator's Excessive Absenteeism or Denying Her Leave Requests. | Does not meet inclusion criteria | 183 | ZELMAN 2019 |
| 580 | Authentic Leaders Creating Healthy Work Environments for Nursing Practice | Does not meet inclusion criteria | 101 | Shirey 2006 |
| 581 | A Narrative Review of Financial Burden, Distress, and Toxicity of Inflammatory Bowel Diseases in the United States | Does not meet inclusion criteria | 2138 | Kahn-Boesel et al., 2023 |
| 582 | Burden associated with chronic sleep maintenance insomnia characterized by nighttime awakenings among women with menopausal symptoms | Does not meet inclusion criteria | 39 | Bolge 2010 |
| 583 | Healthcare Workers' Perceptions of On-Site Childcare | Does not meet inclusion criteria | 2433 | Braddock et al., 2023 |
| 584 | Are preventive measures adequate? An evaluation of the implementation of COVID-19 prevention and control measures in nursing homes in China | Does not meet inclusion criteria | 2595 | Shi et al., 2021 |
| 585 | Exploring compassionate managerial leadership style in reducing employee stress level during COVID-19 crisis: the case of Nigeria. | Does not meet inclusion criteria | 228 | Oruh et al., 2021 |
| 586 | Anxiety, Stress and Loneliness: COVID's Toll on the Lives of Workers | Does not meet inclusion criteria | 146 | N/A |
| 587 | Cost-effectiveness analysis of COVID-19 tests in the unified health system | Does not meet inclusion criteria | 2438 | Vinicius et al., 2023 |
| 588 | The cost-effectiveness of treatment with desloratadine in patients with persistent allergic rhnitis. | Does not meet inclusion criteria | 38 | Sullivan et al., 2010 |
| 589 | Transformational leadership and predictors of resilience among registered nurses: a cross-sectional survey in an underserved area | Does not meet inclusion criteria | 2226 | Salam et al., 2023 |
| 590 | The Potential Health Care Costs and Resource Use Associated with COVID-19 in the United States | Does not meet inclusion criteria | 2617 | Bartsch et al., 2020 |
| 591 | South African business rescue regime: systematic review highlighting shortcomings, recommendations and avenues for future research | Does not meet inclusion criteria | 2461 | Matenda et al., 2023 |
| 592 | Outcomes and process evaluation of a cluster-randomised participatory organisational intervention among German healthcare workers | Does not meet inclusion criteria | 2241 | Montano et al., 2023 |
| 593 | Modelling the impact of non-pharmaceutical interventions on workplace transmission of SARS-CoV-2 in the home-delivery sector | Does not meet inclusion criteria | 2203 | Whitfield et al., 2023 |
| 594 | Developing a smart port architecture and essential elements in the era of Industry 4.0 | Does not meet inclusion criteria | 2587 | Min 2022 |
| 595 | Helping others but Hurting Yourself? The underlying mechanism linking helping behavior to task performance | Does not meet inclusion criteria | 2167 | Bao et al., 2023 |
| 596 | How do job characteristics influence the motivation of millennial hospitality employees? | Does not meet inclusion criteria | 2754 | Seqhobane & Kokt 2021 |
| 597 | Anticipated health effects and proposed countermeasures following the immediate introduction of telework in response to the spread of COVID‐19: The findings of a rapid health impact assessment in Japan | Does not meet inclusion criteria | 2345 | Nagata et al., 2021 |
| 598 | Is Omicron Showing Us the Path Ahead? | Does not meet inclusion criteria | 2403 | El-Sadr & Shea 2022 |
| 599 | Non-modifiable worker and workplace risk factors contributing to workplace absence: A stakeholder-centred synthesis of systematic reviews. | Does not meet inclusion criteria | 226 | White et al., 2015 |
| 600 | The role of dispositional mindfulness in employee readiness for change during the COVID-19 pandemic | Does not meet inclusion criteria | 2622 | Roemer et al., 2021 |
| 601 | Unit-Level Counterproductive Work Behavior (CWB): A Conceptual Review and Quantitative Summary. | Does not meet inclusion criteria | 221 | Carpenter et al., 2021 |
| 602 | Pandemic turned into pademonium: the effect on supply chains and the role of accounting information | Does not meet inclusion criteria | 2627 | Velayutham 2021 |
| 603 | How the sanitation dimensions impacts consumer perceptions and emotions in five-star hotels due to COVID-19 pandemic? | Does not meet inclusion criteria | 2538 | Gupta et al., 2022 |
| 604 | Intelligence: human, economioc and artificial against the effects of Covid-19 | Does not meet inclusion criteria | 2513 | Miró-Pérez & Torrent-Sellens 2020 |
| 605 | The Role of Management Pressure on Employees' Turnover Intention in the Hospitality Industry in Malaysia | Does not meet inclusion criteria | 2660 | Marimuthu & Wahab, 2022 |
| 606 | Employers can save big bucks by offering telecommuting. | Does not meet inclusion criteria | 143 | Eisenberg 2017 |
| 607 | Versatile Bureaucracy: A Telework Case Study. | Does not meet inclusion criteria | 138 | Meadows 2007 |
| 608 | Evaluating the effectiveness of employee assistance programmes: a systematic review. | Does not meet inclusion criteria | 236 | Joseph et al., 2018 |
| 609 | Managing Turnover Strategically. | Does not meet inclusion criteria | 242 | Bluedorn 1982 |
| 610 | Presenteeism in nursing: An evolutionary concept analysis. | Does not meet inclusion criteria | 30 | Rainbow & Steege 2017 |
| 611 | Teleworking Perspectives for Romanian SMEs after the COVID-19 Pandemic | Does not meet inclusion criteria | 2712 | Grigorescu et al., 2020 |
| 612 | Developing policy analytics for public health strategy and decisions - the Sheffield alcohol policy model framework. | Does not meet inclusion criteria | 243 | Brennan et al., 2016 |
| 613 | Covid - 19 Pandemic and its Impact on Labor Force: A New Model Based on Social Stress Theory and Prospect Theory | Does not meet inclusion criteria | 2603 | Manojkrishnan & Aravind, 2020 |
| 614 | Measuring bullying at work with the Short-Negative Acts Questionnaire: Identification of targets and criterion validity. | Does not meet inclusion criteria | 67 | Notelaers et al., 2019 |
| 615 | Menstrual characteristics and dysmenorrhea among Palestinian adolescent refugee camp dwellers in the West Bank and Jordan: a cross-sectional study | Does not meet inclusion criteria | 2246 | Ghandour et al., 2023 |
| 616 | Can worksite nutritional interventions improve productivity and firm profitability? A literature review. | Does not meet inclusion criteria | 36 | Jensen 2011 |
| 617 | The economic burden of coronavirus disease 2019 (COVID-19): evidence from Iran | Does not meet inclusion criteria | 2679 | Mohsen et al., 2021 |
| 618 | An inclusive school for computer science: Evaluating early impact with propensity score matching | Does not meet inclusion criteria | 2215 | Corning et al., 2023 |
| 619 | Editorial: Hard facts or half-truths? The social and economic sustainability impact of flexible work practices in organizations | Does not meet inclusion criteria | 2164 | Aboagye et al, 2023 |
| 620 | Two in Distress Make Sorrow less: A Work-Life Conflict Moderated Mediation Model | Does not meet inclusion criteria | 2740 | Martínez-Corts et al., 2021 |
| 621 | IN BRIEF | Does not meet inclusion criteria | 2311 | Anonymous 2020 |
| 622 | Behavioral health and disability insurance: A perspective. | Does not meet inclusion criteria | 77 | Mitchell 2011 |
| 623 | Menstrual hygiene practice and associated factors among adolescent girls in sub-Saharan Africa: a systematic review and meta-analysis | Does not meet inclusion criteria | 2216 | Anbesu & Asgedom, 2023 |
| 624 | Absenteeism in organizations: Approaches to identify determinants and suggestions for interventions to promote health at the workplace. | Does not meet inclusion criteria | 112 | Kleinbeck et al., 1996 |
| 625 | Malaysia Pharmaceuticals Report - Q1 2023 | Does not meet inclusion criteria | 269 | Statista |
| 626 | Examining the impact of reasonable accommodation appraisals on New Zealand managers' attitudes toward hiring people with disability | Does not meet inclusion criteria | 2163 | D'Souza & Kuntz 2023 |
| 627 | Obesity and injury-related absenteeism in a population-based firefighter cohort. | Does not meet inclusion criteria | 89 | Poston et al., 2011 |
| 628 | Working with influenza-like illness: Presenteeism among US health care personnel during the 2014-2015 influenza season. | Does not meet inclusion criteria | 59 | Chiu et al., 2017 |
| 629 | EXPLORING PERCEPTIONS of U.S. Healthcare & Public Safety Workers at the Onset of the COVID-19 Pandemic | Does not meet inclusion criteria | 2552 | Haas et al., 2022 |
| 630 | Study Released on Office Health Hazards. | Does not meet inclusion criteria | 253 | N/A |
| 631 | Sectoral changes of employment in Poland during the COVID-19 pandemic: Are reallocation shock effects applicable? | Does not meet inclusion criteria | 2496 | Kwiatkowski & Szymańska, 2022 |
| 632 | Wellness and productivity perceptions of general and special education high school teachers: A case study. | Does not meet inclusion criteria | 65 | Gilbert 2020 |
| 633 | Intersectoral costs of sexually transmitted infections (STIs) and HIV: a systematic review of cost-of-illness (COI) studies | Does not meet inclusion criteria | 2321 | Schnitzler et al., 2021 |
| 634 | Workplace Resilience and Performance: Workload and Organizational Constraints | Does not meet inclusion criteria | 2725 | Mallak & Shank 2021 |
| 635 | "More Than Three Consecutive Days" Means 72 Hours; DOL's Regulation Is Valid Under FMLA. | Does not meet inclusion criteria | 239 | U.S. Department of Labour |
| 636 | Lives Put on Hold: The Impact of the COVID-19 Pandemic on Canada's Youth | Does not meet inclusion criteria | 2531 | Mahboubi 2022 |
| 637 | Antecedents and consequences of employee engagement in the SMEs of crafts | Does not meet inclusion criteria | 2735 | Istiningsih et al., 2020 |
| 638 | Why we need to talk about 'PPE' for mental health | Does not meet inclusion criteria | 2337 | Morley 2020 |
| 639 | TERMINATING CHRONICALLY ABSENT EMPLOYEES. | Does not meet inclusion criteria | 213 | Côté 2008 |
| 640 | Effects of Slide Sheet Use and Bed Position on Muscle Activities in the Low Back and Extremities: A Pilot Experimental Simulation Study | Does not meet inclusion criteria | 2139 | Higuchi et al., 2023 |
| 641 | Developing a Multilevel Scale to Assess Retention of Workers with Disabilities | Does not meet inclusion criteria | 2506 | KrisiMiri et al., 2022 |
| 642 | From Conexa to Docpass: The Competitive Environment of Telemedicine Platforms | Does not meet inclusion criteria | 2665 | Moreira et al., 2021 |
| 643 | COVID-19 HITS THE FRENCH HEALTH SYSTEM | Does not meet inclusion criteria | 2612 | Jones 2020 |
| 644 | How is COVID-19 pandemic causing employees withdrawal behaviour in the hospitality industry? An empirical investigation | Does not meet inclusion criteria | 2402 | Khawaja et al., 2022 |
| 645 | The Benefits and Costs of Using Social Distancing to Flatten the Curve for COVID-19 | Does not meet inclusion criteria | 2758 | Thunström et al., 2020 |
| 646 | Effects of automated communication on team members' activity and social presence awareness, commitment, and motivation in human-autonomy teams | Does not meet inclusion criteria | 2214 | Graf et al., 2023 |
| 647 | MANAGEMENT DURING CRISES: THE CASE OF THE EAPN ACTION ON THE NORTH REGION OF PORTUGAL | Does not meet inclusion criteria | 2635 | Leite et al., 2021 |
| 648 | Multiple control mechanisms for employee health and safety integration: effects and complementarity | Does not meet inclusion criteria | 2703 | Passetti et al., 2020 |
| 649 | TROIS QUESTIONS À . . . | Does not meet inclusion criteria | 144 | CHARPENTIER 2023 |
| 650 | A COVID-19 contextual study of customers' mistreatment and counterproductive work behavior at coffee cafés | Does not meet inclusion criteria | 2489 | Ahmed et al., 2021 |
| 651 | Could 2021 be the year OH seizes its moment? | Does not meet inclusion criteria | 2356 | Paton 2021 |
| 652 | Learning to work from home: experience of Australian workers and organizational representatives during the first Covid-19 lockdowns | Does not meet inclusion criteria | 2362 | Marzban et al., 2021 |
| 653 | The physical, emotional and behavioral symptoms of health problems among employees before and during the COVID-19 epidemic | Does not meet inclusion criteria | 2434 | Rožman & Tominc, 2022 |
| 654 | A systematic review of infectious illness Presenteeism: prevalence, reasons and risk factors. | Does not meet inclusion criteria | 54 | Webster et al., 2019 |
| 655 | ADAPTABILITY TO TELEWORKING IN EUROPEAN COUNTRIES | Does not meet inclusion criteria | 2361 | Bălăcescu et al., 2021 |
| 656 | Pandemics at Work: Convergence of Epidemiology and Ethics | Does not meet inclusion criteria | 2408 | Thornton & Martin 2022 |
| 657 | Municipal Responses to COVID-19: the case of library closures in New South Wales local government | Does not meet inclusion criteria | 2658 | Wallace & Dollery, 2021 |
| 658 | COVID-19 Emergency Sick Leave Has Helped Flatten The Curve in the United States | Does not meet inclusion criteria | 2371 | Pichler et al., 2020 |
| 659 | Content Validity and Psychometric Evaluation of the Functional Assessment of Chronic Illness Therapy–Fatigue (FACIT–Fatigue) in Patients with Crohn’s Disease and Ulcerative Colitis | Does not meet inclusion criteria | 260 | Loftus et al., 2023 |
| 660 | Consumer Intention towards Blended Learning a Mediating Role of Attitude | Does not meet inclusion criteria | 2620 | Verma et al., 2022 |
| 661 | Labor shortage: a critical reflection and a call for industry-academia collaboration | Does not meet inclusion criteria | 2469 | Kwok 2022 |
| 662 | Managerial Emotional Intelligence and Organizational Survival of Government Hospitals in Delta State of Nigeria | Does not meet inclusion criteria | 2763 | Henry & Amah 2021 |
| 663 | The effect of personalized intelligent digital systems for self-care training on type II diabetes: a systematic review and meta-analysis of clinical trials | Does not meet inclusion criteria | 2135 | Tanhapour et al., 2023 |
| 664 | Unpacking strategic corporate social responsibility in the time of crisis: a critical review | Does not meet inclusion criteria | 2487 | Ashraf et al., 2022 |
| 665 | Using Economics to Impact Local Obesity Policy: Introducing the UK Centre for Economics of Obesity (CEO) | Does not meet inclusion criteria | 2624 | Frew et al., 2022 |
| 666 | The impact of leadership on perceived work-related stress in healthcare facilities organisations | Does not meet inclusion criteria | 2576 | Greco et al., 2022 |
| 667 | Active surveillance for acute respiratory infections among pediatric long-term care facility staff | Does not meet inclusion criteria | 119 | Wilmont et al., 2020 |
| 668 | Cost of medication overuse headache in Italian patients at the time-point of withdrawal: a retrospective study based on real data | Does not meet inclusion criteria | 29 | D'Amico et al., 2017 |
| 669 | Enterprises' perception and practice of humane entrepreneurship | Does not meet inclusion criteria | 2521 | Dębicka et al., 2022 |
| 670 | How to cut absenteeism. | Does not meet inclusion criteria | 204 | Fowler 1998 |
| 671 | Managing bullying in the workplace: a model of servant leadership, employee resilience and proactive personality | Does not meet inclusion criteria | 2692 | Ahmad et al., 2021 |
| 672 | Another Look at Attendance Control. | Does not meet inclusion criteria | 194 | Thomas 1968 |
| 673 | Self-efficacy in the context of organizational psychology. | Does not meet inclusion criteria | 102 | Martincevic 2004 |
| 674 | What innovations would enable hospitality in South Africa to rebuild? | Does not meet inclusion criteria | 2387 | Nyathela-Sunday et al., 2022 |
| 675 | Contingency Theory, Values, and Change. | Does not meet inclusion criteria | 249 | Millar 1978 |
| 676 | Mapping the Sustainable Human-Resource Challenges in Southeat Asia's FinTech Sector | Does not meet inclusion criteria | 2619 | An-Chi & Duc-Dinh 2022 |
| 677 | An evening of alcohol consumption negatively impacts next-day immune fitness in both hangover-sensitive drinkers and hangover-resistant drinkers | Does not meet inclusion criteria | 2105 | Merlo et al., 2023 |
| 678 | Addressing the second victim phenomenon in Israeli health care institutions | Does not meet inclusion criteria | 2219 | Cohen & Nissanholtz-Gannot 2023 |
| 679 | Gender equality and comparative HRM: A 40-year review | Does not meet inclusion criteria | 2234 | Garcia 2023 |
| 680 | Physician Leadership Around the World | Does not meet inclusion criteria | 2324 | Anonymous 2022 |
| 681 | TIME OUT | Does not meet inclusion criteria | 207 | Penttila 2005 |
| 682 | Leadership and strategy in the news | Does not meet inclusion criteria | 2739 | Henry 2021 |
| 683 | College Students' Autonomous Learning Behavior Based on Big Data and Internet of Things. | Does not meet inclusion criteria | 212 | Hong 2023 |
| 684 | Medicalization of sickness absence. | Does not meet inclusion criteria | 91 | Schlerenbeck 2010 |
| 685 | Health Services and Patient Satisfaction in IRAN during the COVID-19 Pandemic: A Methodology Based on Analytic Hierarchy Process and Artificial Neural Network | Does not meet inclusion criteria | 2565 | Khansari et al., 2022 |
|  | **Full-Text Screening** | | | |
| 686 | Managing COVID-19 in workplaces - a theoretical framework for integral employees' health | Secondary Study | 2575 | Gauer 2022 |
| 687 | Absenteeism | Wrong study design | 180 | N/A |
| 688 | Multi-grade fuzzy assessment framework for software professionals in work-from-home mode during and post-COVID-19 era | The study is not about presenteeism | 2560 | Suresh 2021 |
| 689 | Perceived legitimacies of health-related and motivational presenteeism and absenteeism: Development and validation of the Workplace Attendance Behavior Legitimacy Scale | Study not about post covid telework | 2122 | Ruhle 2023 |
| 690 | Resilience, well-being, and organizational outcomes of Croatian, Thai, and US workers during COVID-19 | No indication of at least 50% telework | 2376 | Charoensap-Kelly 2021 |
| 691 | The high cost of 'presenteeism' | Wrong study design | 211 | N/A |
| 692 | Economic burden of chronic migraine in OECD countries: a systematic review | Wrong study design | 258 | Eltrafi 2023 |
| 693 | The influence of workplace happiness and innovative work behavior on job satisfaction mediated by work engagement | Study not about post covid telework | 2714 | Rosdaniati 2021 |
| 694 | Excessive availability for work: Good or bad? Charting underlying motivations and searching for game-changers. | Study not about post covid telework | 82 | Cooper 2019 |
| 695 | The COVID-19 office in transition: cost, efficiency and the social responsibility business case | Wrong study design | 2598 | Parker 2020 |
| 696 | Mental health experiences of HIV/TB healthcare workers during the COVID-19 pandemic – lessons for provider well-being and support from a qualitative study in seven South African provinces | Study not about post covid telework | 2399 | Yang 2023 |
| 697 | Mandates Narrow Gender Gaps In Paid Sick Leave Coverage For Low-Wage Workers In The US | The study is not about presenteeism | 2318 | Harknett 2022 |
| 698 | Organization Development and Agility During Economic and Operational Uncertainty. | Don't define | 147 | Ballaro 2022 |
| 699 | What's Trending For Employee Wellness In 2022? | Wrong study design | 162 | Lagemann 2021 |
| 700 | Telework and Work–Family Conflict during COVID-19 Lockdown in Portugal: The Influence of Job-Related Factors | The study is not about presenteeism | 2760 | Andrade 2021 |
| 701 | Wellbeing or Workaholism - Critical Perspectives on Employee Engagement in New Work Practices. | Study not about post covid telework | 251 | Shaik 2019 |
| 702 | Examining Telecommuting During Pandemic in Malaysia: Is There Support? | The study is not about presenteeism | 2359 | Jais 2022 |
| 703 | Digital labor of school teachers and their well-being: content analysis of English-language dailies in India | The study is not about presenteeism | 2495 | Priya 2022 |
| 704 | How modern working environments shape attendance behaviour: A longitudinal study on weekly flexibilization, boundaryless work and presenteeism | Study not about post covid telework | 2182 | Poethke 2023 |
| 705 | Working from home increases presenteeism problems for employers. | Wrong study design | 132 | Simon 2020 |
| 706 | Price of presenteeism. | Wrong study design | 121 | Juniper 2012 |
| 707 | Do digital literacies matter in employee engagement in digitalised workplace? | The study is not about presenteeism | 2306 | Chan 2021 |
| 708 | Understanding Digital Work and its Use in Organizations from a Literature Review | Wrong study design | 2646 | Santoso Wibowo 2022 |
| 709 | Identification of Key Psychosocial Safety Factors when Working Remotely: A Three-Step Research Methodology Proposal | The study is not about presenteeism | 2744 | Gazdecka 2021 |
| 710 | Work Overload and Self-Endangering Work Behavior: The Amplifying and Buffering Role of Work Autonomy and Self-Leadership | The study is not about presenteeism | 2196 | Mander 2023 |
| 711 | The Effects of a Mandatory Work from Home Policy on Respect, Trust, and Mutual Obligations During the COVID-19 Pandemic in Switzerland | The study is not about presenteeism | 2567 | Schreier 2022 |
| 712 | Supporting nurses and their mental health in a world after Covid-19 | Wrong study design | 2327 | Teoh 2020 |
| 713 | Is teleworking at odds with social sustainability and organizational learning? | The study is not about presenteeism | 2497 | Mosquera 2022 |
| 714 | A Cross-Sectional Study of the Association Between Telecommuting Environments and Shoulder Pain Among Japanese Telecommuting Workers | The study is not about presenteeism | 2193 | Sato 2023 |
| 715 | Enforced remote working and the work-life interface during lockdown | Wrong study design | 2382 | Anderson 2020 |
| 716 | In Absence of Absenteeism: Some Thoughts on Productivity Costs in Economic Evaluations in a Post-corona Era. | No indication of at least 50% telework | 163 | Brouwer 2022 |
| 717 | LE TÉLÉTRAVAIL FACE AU « BIAIS DE PROXIMITÉ ». | Not in English | 129 | WOLSKI 2022 |
| 718 | Associations of working from home with occupational physical activity and sedentary behavior under the COVID‐19 pandemic | The study is not about presenteeism | 2380 | Fukushima 2021 |
| 719 | Burnt to a crisp? Understanding drivers of burnout amongst New Zealand workers | Study not about post covid telework | 2574 | Haar 2022 |
| 720 | Working from home: Absence makes the heart grow fonder. | Wrong study design | 141 | N/A |
| 721 | Mindfulness, remote engagement and employee morale: conceptual analysis to address the “new normal” | Wrong study design | 2707 | Pattnaik 2021 |
| 722 | Working during the COVID-19 pandemic: Demands, resources, and mental wellbeing | Don't define | 2301 | Scheel 2023 |
| 723 | Two definitions of presenteeism: sickness presenteeism and impaired work function | Study not about post covid telework | 2 | Ishimaru 2020 |
| 724 | Musculoskeletal pain among teleworkers: Frequency and associated factors | The study is not about presenteeism | 2168 | Fiorini 2023 |
| 725 | Stress in the Working Environment and its Causes | Secondary Study | 2449 | Pfejfer-Buczek 2023 |
| 726 | Work and health-related factors of presenteeism: a mediation analysis on the role of menopausal symptoms between job demands and presenteeism among a sample of social service women employees | Study not about post covid telework | 277 | Guidetti 2022 |
| 727 | Italians and smart working: A technical study on the effects of smart working on the society | Don't define | 2180 | Fortuna 2023 |
| 728 | Concerns, quality of life, access to care and productivity of the general population during the first 8 weeks of the coronavirus lockdown in Belgium and the Netherlands | Study not about post covid telework | 2351 | Hannevan Ballegooijen 2021 |
| 729 | Post COVID-19 condition, work ability and occupational changes in a population-based cohort | No indication of at least 50% telework | 2110 | Kerksieck 2023 |
| 730 | Barriers and facilitators of productivity while working from home during pandemic | The study is not about presenteeism | 2323 | Seva 2021 |
| 731 | An Assessment of the Effect of Work from Home on Academic Worker's Well-Being in Malaysian Higher Educational Institutions | The study is not about presenteeism | 2516 | Sulaiman 2022 |
| 732 | CURRENT SITUATION AND PERSPECTIVE REGARDING TELEWORK IN THE PERCEPTION OF MANAGERS FROM SMALL AND MEDIUMSIZED ENTERPRISES | The study is not about presenteeism | 2353 | Budacia 2021 |
| 733 | A prospective cohort study of presenteeism and poverty among Japanese workers during the COVID‐19 pandemic | Study not about post covid telework | 2328 | Fujino 2022 |
| 734 | Working in Public Administration During Nonwork Time During the COVID-19 Pandemic | The study is not about presenteeism | 2642 | Mar. 2021 |
| 735 | Exhaustion while teleworking during COVID-19: a moderated-mediation model of role clarity, self-efficacy, and task interdependence | Don't define | 2648 | Mihalca 2021 |
| 736 | A Qualitative Study of Pandemic-Induced Telework: Federal Workers Thrive, Working Parents Struggle | Unable to locate the full-text | 2466 | Mullins 2022 |
| 737 | Sick Days Don't Look So Good Now That You Can Work From Home. | Wrong study design | 131 | Carmichael 2021 |
| 738 | Real work at home. | Secondary Study | 166 | Davies 2007 |
| 739 | Work from home practices as corporate strategy- an integrative review | The study is not about presenteeism | 2161 | Agrawal 2023 |
| 740 | Employee presenteeism and occupational acquisition of COVID-19. | Study not about post covid telework | 50 | Eisen 2020 |
| 741 | Home Is Where The Office Is. | Wrong study design | 177 | Phelan 2002 |
| 742 | Work from Home: Measuring Satisfaction between Work–Life Balance and Work Stress during the COVID-19 Pandemic in Indonesia | The study is not about presenteeism | 2690 | Irawanto 2021 |
| 743 | Theoretical and Empirical Advancements in Presenteeism Research. | Wrong study design | 135 | Breitsohl 2022 |
| 744 | Centrality of psychological well-being of IT employees during COVID-19 and beyond | Don't define | 2463 | Gupta 2022 |
| 745 | Presenteeism: A review and research directions. | Study not about post covid telework | 69 | Lohaus 2019 |
| 746 | Home-Based Telework and Sickness Presenteeism. | Wrong study design | 15 | Kawada 2021 |
| 747 | Sitting for long periods is associated with impaired work performance during the COVID‐19 pandemic | Don't define | 2315 | Wakaizumi 2021 |
| 748 | Rebuilding the workplace to promote young workers' mental health | No indication of at least 50% telework | 2338 | Parry 2022 |
| 749 | Working from Home During the COVID-19 Pandemic: Implications for Workplace Relationships 1 | The study is not about presenteeism | 2372 | Espersson 2023 |
| 750 | Satisfaction with Work-Life Balance During the COVID-19 Pandemic for Full-Time Workers Forced to Work from Home | The study is not about presenteeism | 2446 | Mathis 2023 |
| 751 | Associations between job and workplace factors, health and physical factors, personal factors, and presenteeism among general employees in Japan: A longitudinal study | No indication of at least 50% telework | 276 | Goto 2022 |
| 752 | Associations Between Telework Experience and Psychosocial Working Conditions During the COVID-19 Pandemic: A Cross-sectional Analysis Among White-Collar Workers in Sweden | The study is not about presenteeism | 2159 | Svensson 2023 |
| 753 | Working from home: small business performance and the COVID-19 pandemic | The study is not about presenteeism | 2391 | Zhang 2022 |
| 754 | Remote Work in Peru during the COVID-19 Pandemic | Study not about post covid telework | 2405 | Alexander Frank Pasquel Cajas 2023 |
| 755 | Impact of Telecommuting on Clinician Absenteeism in Primary Care-Potential Solution for Burnout?. | Secondary Study | 20 | Caron 2021 |
| 756 | Unlock the black box of remote e-working effectiveness and e-HRM practices effect on organizational commitment | Don't define | 2650 | Alshibly 2022 |
| 757 | The perceived influence of remote working on specific human resource management outcomes during the COVID-19 pandemic | Secondary Study | 2502 | Saurombe 2022 |
| 758 | Hope during the COVID-19 lockdown – the role of organization interventions | The study is not about presenteeism | 2462 | Wickramasinghe 2023 |
| 759 | The monitoring of tele-homeworkers in the UK: legal and managerial implications | The study is not about presenteeism | 2699 | Lockwood 2021 |
| 760 | "To work, or not to work, that is the question" – Recent trends and avenues for research on presenteeism. | Secondary Study | 196 | Ruhle 2020 |
| 761 | The consequences of sickness presenteeism on health and wellbeing over time: A systematic review. | Secondary Study | 32 | Skagen 2016 |
| 762 | Is working from home the new workplace panacea? Lessons from the COVID-19 pandemic for the future world of work | The study is not about presenteeism | 2577 | deKlerk 2021 |
| 763 | Trajectories of Teleworking via Work Organization Conditions: Unraveling the Effect on Work Engagement and Intention to Quit with Path Analyses | The study is not about presenteeism | 2262 | Parent-Lamarche 2023 |
| 764 | Remote workers' experiences with electronic monitoring during Covid-19: implications and recommendations | Wrong study design | 2331 | Jeske 2022 |
| 765 | Examining staff burnout during the transition to teaching online due to COVID-19 implications | Don't define | 2410 | Fynn 2023 |
| 766 | Telework's impact on employee effectiveness: Is it the time or the place that really matters? | Study not about post covid telework | 72 | Alexander 2016 |
| 767 | Professional isolation and pandemic teleworkers’ satisfaction and commitment: The role of perceived organizational and supervisor support | Wrong study design | 2277 | Deschênes 2023 |
| 768 | Digital Leadership as a Resource to Enhance Managers' Psychological Well-Being in the COVID-19 Pandemic Situation in Indonesia | The study is not about presenteeism | 2687 | Dewi 2021 |
| 769 | Influence of Remote Work on the Work Stress of Workers in the Context of the COVID-19 Pandemic: A Systematic Review | Wrong study design | 2133 | Dávila Morán 2023 |
| 770 | COVID-19 and the reimagining of working while sick | Secondary Study | 2309 | Johnson 2021 |
| 771 | Supporting Knowledge Workers’ Health and Well-Being in the Post-Lockdown Era | Study not about post covid telework | 2346 | Harkiolakis 2023 |
| 772 | Investigating the Impact of Remote Working on Employee Productivity and Work-life Balance: A Study on the Business Consultancy Industry in Dubai, UAE | Wrong study design | 2535 | Rañeses 2022 |
| 773 | Development and Implementation of Work Engagement Strategies in a Clinical Research Consortium During the Coronavirus Disease 2019 (COVID-19) Pandemic: A Reflective Inquiry | Wrong study design | 2736 | Johnson 2021 |
| 774 | Behavioral Interventions to Improve Home-Based Office-Workers’ Health | Wrong study design | 2184 | Bartmann 2023 |
| 775 | medical incapacity and absenteeism. | Unable to locate the full-text | 203 | Hoyle 2005 |
| 776 | Effect of occupational stress and remote working on psychological well-being of employees: an empirical analysis during covid-19 pandemic concerning information technology industry in hyderabad | Study not about post covid telework | 2709 | Prasad 2020 |
| 777 | Is Online Teaching Challenging Faculty Well-Being? | The study is not about presenteeism | 2555 | Mosquera 2022 |
| 778 | sickout. | Wrong study design | 198 | N/A |
| 779 | Hindering and enabling factors for young employees with common mental disorder to remain at or return to work affected by the Covid-19 pandemic – a qualitative interview study with young employees and managers | The study is not about presenteeism | 2183 | Mosquera 2022 |
| 780 | Antecedents and consequences of telecommuting on public organizations in Iran: the case of west Azerbaijan province | Unable to locate the full-text | 2171 | Bashir Khodaparasti 2023 |
| 781 | A Study of the Remote Work-Family Balance of Female Hotel Managers | Study not about post covid telework | 2173 | Bhatt 2023 |
| 782 | Exploring the effects of remote work on employee productivity in Botswana amidst the COVID-19 Pandemic | The study is not about presenteeism | 2426 | Chiguvi 2023 |
| 783 | Promoting remote workers' psychological health: Effective management practices during the COVID-19 crisis | Don't define | 2162 | Bouchard 2023 |
| 784 | Presenteeism behaviour: Current research, theory and future directions. | Secondary Study | 64 | McGregor 2022 |
| 785 | The impact of work engagement and meaningful work to alleviate job burnout among social workers in New Zealand | The study is not about presenteeism | 2427 | Kim-Lim and Peik 2022 |
| 786 | A Framework for the Community Psychiatrist’s Role in the COVID-19 Response | Wrong study design | 2551 | Fetter 2021 |
| 787 | Trauma Data Quality Improvement: One Center's Experience With Telecommuting and Paperless Data Management. | The study is not about presenteeism | 21 | Seegert et al. 2020 |
| 788 | MANAGERIAL AGILITY AND TELEWORKING IN TIMES OF CRISIS - COVID-19: THE CASE OF THE MULTINATIONAL WEBHELP | Secondary Study | 2529 | El Abidine and Samira 2022 |
| 789 | Association between work style and presenteeism in the Japanese service sector. | Study not about post covid telework | 16 | Ishimaru and Fujino 2021 |
| 790 | The relationship between homeworking during COVID-19 and both, mental health, and productivity: a systematic review | Wrong study design | 2202 | Hall et al., 2023 |
| 791 | Shedding light on the work burden of long COVID | Wrong study design | 2141 | Lemogne and Pitron, 2023 |
| 792 | Changes in Job Situations for Women Workforce in Construction during the COVID-19 Pandemic | Study not about post covid telework | 2367 | Bee and Benson 2021 |
| 793 | Sick and working: Current challenges and emerging directions for future presenteeism research | Secondary Study | 2177 | Patel et al., 2023 |
| 794 | If and to What Extent Does Organizational Learning Culture Predict Turnover Intentions of Telecommuting Call Centre Agents? | Secondary Study | 2394 | Pinzon et al., 2023 |
| 795 | For Telework, Please Dial 7 - Qualitative Study on the Impacts of Telework on the Well-Being of Contact Center Employees during the COVID-19 Pandemic in Portugal | Don't define | 2460 | Reinaldo & Silvia, 2023 |
| 796 | Treatment interruption is a risk factor for sickness presenteeism: A large-scale cross-sectional study during the COVID-19 pandemic | No indication of at least 50% telework | 2308 | Okawara et al., 2022 |
| 797 | Teleworking and Remote Work in Local Government Administration Management in Poland | Secondary Study | 2729 | Goździewska-Nowicka et al., 2020 |
| 798 | THE IMPACT OF HYBRID WORKPLACE MODELS ON INTANGIBLE ASSETS: THE CASE OF AN EMERGING COUNTRY | Don't define | 2675 | Radonić et al., 2021 |
| 799 | Digital Stress. Effects of Different Intensities of Working from Home on Workers' Health | Don't define | 2175 | Rodríguez-Modroño 2023 |
| 800 | "Whenever I can I push myself to go to work": a qualitative study of experiences of sickness presenteeism among workers with rheumatoid arthritis | Study not about post covid telework | 57 | Holland and Collins 2018 |
| 801 | Occupational Helath Science in the Time of COVID-19: Now more than Ever. | Wrong study design | 11 | Sinclair et al., 2020 |
| 802 | Examining the effects of remote work arrangements implemented during the COVID-19 pandemic on the overall wellness of employees at Botswana Unified Revenue Services (BURS) | The study is not about presenteeism | 2478 | Chiguvi et al., 2023 |
| 803 | Presenteeism is Reloaded and Ready for Further Research - A Commentary on Priebe, J. and Hagerbaumer, M. (2023) | Wrong study design | 2127 | Breitsohl et al., 2023 |
| 804 | The Impact of Covid-19 on Employees' Work-Life Balance: A Case Study of Botswana Unified Revenue | The study is not about presenteeism | 2390 | Chiguvi et al., 2023 |
| 805 | Rethinking the Unthinkable: A Delphi Study on Remote Work during COVID-10 Pandemic | The study is not about presenteeism | 2181 | Galanti et al., 2023 |
| 806 | _COVID-19 Emergency Sick Leave Has Helped Flatten The Curve In the United States | Study not about post covid telework | 2320 | Pichler et al., 2020 |
| 807 | Home-Based Telework and Presenteeism Across Europe. | Wrong Study Design | 17 | Steidelmuller 2020 |
| 808 | Sickness presenteeism: Are we sure about what we are studying? A research based on a literature review and an empirical illustration. | Wrong Study Design | 3 | Navarro 2019 |
| 809 | The Role of Impaired Recovery in Negative Consequences of Workplace Telepressure. | Wrong Study Design | 81 | Hu 2019 |

**Table 3:** Legend

| **Reason for Exclusion** | **Total Number of Articles** | **Explanation of Reasons** |
| --- | --- | --- |
| Does not meet inclusion criteria | 685 | Total number of articles excluded throughout screening process (i.e. at the title/abstract level of screening and full-text level of screening) |
| The study is not about presenteeism | 36 | Does not meet inclusion criteria |
| Don't define | 12 | No clear definition of presenteeism and/or absenteeism |
| Secondary study | 13 | Does not meet inclusion criteria |
| Wrong study design | 31 | Does not meet inclusion criteria |
| Study not about post covid telework | 22 | Does not meet inclusion criteria |
| Unable to locate full-text | 3 | Does not meet inclusion criteria |
| Not in English | 1 | Does not meet inclusion criteria |
| No indication of at least 50% telework | 6 | Does not meet inclusion criteria |
| **Total Excluded Articles** | **809** |  |
