# Supplementary material for: Sick leave or work sick? Examining the antecedents and conceptualizations of presenteeism and absenteeism among teleworkers during COVID-19: A scoping review
Source: PLOS Ment Health. 2025 May 13;2(5):e0000300. doi: 10.1371/journal.pmen.0000300 (PMC12798164; doi:10.1371/journal.pmen.0000300)
Supplement: Attachment — Submitted filename: Response_to_Reviewers_auresp_3.docx [file pmen.0000300.s006.docx]

**Response to Reviewers**

**Journal Requirements**

**Comment #1:** Please review your reference list to ensure that it is complete and correct. If you have cited papers that have been retracted, please include the rationale for doing so in the manuscript text or remove these references and replace them with relevant current references. Any changes to the reference list should be mentioned in the rebuttal letter that accompanies your revised manuscript. If you need to cite a retracted article, indicate the article’s retracted status in the References list and also include a citation and full reference for the retraction notice.

**Response to Comment #1:** Thank you for your comment. We have indicated in our manuscript that we further excluded three articles after data extraction as they fall outside of our timeframe (prior to 2020). The articles that we excluded were Hu et al, 2019; Navarro et al. 2019; and Steidelmuller et al. 2020.

**Reviewer 2 Comments**

**Comment #1:** As with my first review, I hope NOT to deter the authors from making further revisions in order to make their manuscript (in my opinion) publishable, as the subject matter remains valuable and helps fill a knowledge gap. I also acknowledge the efforts put in to respond to the first review and the re-review, but the impression gained is that the second revision of the manuscript was rushed and not sufficiently thorough. Thus, many of the same concerns remain regarding typographical errors, tense inconsistencies, formatting and grammatical errors. There is now also a new set of concerns introduced by the revision of the data corpus from 21 to 18, given that this has not been dealt with systematically throughout the revised manuscript – thus, obvious errors now appear in the Results section.

**Response to Comment #1:** Thank you for your comment. We have reviewed the manuscript to ensure consistent grammar.

**Comment 2:** In the Introduction and Methods section, it is concerning that the authors themselves seem not to have thought through the timeframe of their review fully enough to realize from the outset that studies published before 2020 could not, by definition, have dealt with the COVID-19 pandemic period which was only declared in 2020. Nonetheless, it is appreciated that they have now seen fit to re-consider their inclusion criteria and strip another three studies out of their analysis. However, rather than doing a careful reworking of their Abstract and Results and Discussion sections, their revision seems rather piecemeal and non-thorough. Their ongoing mentions of 2019 create confusion and need to be reconsidered, eg.

• Lines 145-146: “...studies that examined telework during the COVID-19 pandemic (2019 and onwards)” [my emphasis].
• Lines 155-156: “...studies that examined a move to telework arrangements before 2019”. [my emphasis – surely this should be “before the pandemic’s onset” or at least “before 2020”?]
• Lines 167-168: “We included studies published from January 2019 to December 2023 168 (inclusive) to fully capture the impacts of the COVID-19 pandemic”. [this timeframe needs to change – all pre-pandemic studies were excluded in the revised set]
• Last inclusion criterion in Table 1
• Last exclusion criterion in Table 1

**Response to Comment #2:** Thank you for your comment. We have revised the manuscript to indicate the inclusion of studies from 2020 onwards.

**Comment 3:** In the PRISMA chart, the “wrong timeframe” needs to be corrected from n=22 to n=25 for all the numbers to make sense.

**Response to Comment #3:** Thank you for the suggestion. We have revised the PRISMA chart so that the number of studies that failed to meet our timeline is 25 as opposed to 22.

**Comment 4:** In the Results section there are various changes needed. The table of included studies needs its heading to be corrected (it still says ‘n=21’). It also does not follow the usual convention of those studies appearing consecutively in the reference list – instead, the reference numbers for the studies jump around in a confusing manner. It would be preferable to follow convention, although this will require the authors to put significant time into rejuggling their citations throughout the paper and finalising the reference list. Then, with regards to their statistical analyses, the authors need firstly to search for and remove all references to a data corpus of 21 studies and replace those with mentioning of 18 studies. Then they need to recheck their calculations in every respect and must make absolutely certain that none of their key findings disappeared or changed when the three studies were belatedly excluded.

**Response to Comment #4:** Thank you for the comments. We have looked over the manuscript and revised the number of studies as well as its calculations.

Some examples of errors in the Results section include:
**Comment 5:** Lines 256-257: it makes no sense to say “...with the other 19 (90.5%) having a single country focus [1,2,17,19,25,38–50]”. Please re-check exactly which studies should be cited here and recalculate the percentage out of 18. Likewise, please re-check the figures and citations in the rest of that paragraph (up to and including line 264).

**Response to Comment #5:** Thank you for your comment. We have fixed the numbers in the manuscript to reflect the number of studies that were included in the final manuscript.

**Comment 6:** In the paragraph starting with line 265 the figures and especially percentages need to be re-checked and corrected. For instance, 16 out of 18 studies equates to 89%, not 76%, and the authors should double-check that all the studies cited were from their revised data corpus. Similarly, line 266 includes the nonsensical wording “...nine (56.3%) of those being cross-sectional [19, 38,40,42,44,45,47,48]” when only eight studies are cited; furthermore, nine out of 18 studies would equate to 50%, not 56.3%.

**Response to Comment #6:** Thank you for your comment. We have revised the numbers in the manuscript accordingly.

**Comment 7:** In lines 271-272 there is also some immediately apparent incorrect information, where the authors say “Most of the studies (18; 86%) provided gender breakdowns...” – from a cursory check of the (revised) table of included studies, that wording needs to change to “All of the studies (18; 100%) provided gender breakdowns.” This then makes the sentence on lines 273-274 redundant so it can be removed altogether: “However, of the total participant set of 54,457 across all study samples, no gender was specified for 14,058 (26%)”.

**Response to Comment #7:** Thank you for the comment. We have revised the sentence accordingly and removed lines 273-274 to avoid redundancy.

**Comment 8:** The authors need to search for and remove any references to the previous participant set of 54,457 – the revised data corpus contained a participant set of only 27,185.

**Response to Comment #8:** Thank you for your comment, we have changed 54,457 participants to 27,185.

**Comment 9:** Line 254: “Majority of the studies were from European and North American continents (n = 18)” – since the revised dataset was 18 studies, the authors should check whether this should read “All the studies (18;100%)...”.

**Response to Comment #9:** Thank you for the comment. We have revised inconsistencies with the number of studies included.

**Comment 10:** Lines 270-273 are no longer correct.

**Response to Comment #10:** Thank you for your comment. The paragraph has been revised to reflect the included studies.

**Comment 11:** Line 281 – please recalculate the percentage: 17 out of 18 studies equates to 94%, not 81%. Also, please check that the correct studies have been cited.

**Response to Comment #11:** Thank you for your comment. We have fixed the calculations for this section.

**Comment 12:** Lines 282-284 – again, the authors should check that the correct studies have been cited; they include ref [42] but that is one of the studies excluded in Table 2.

**Response to Comment #12:** Thank you for your comment. Reference 42 is part of our included studies (Mauricio and Laranjeira).

**Comment 13:** Line 290 – “four (19%) of the studies...” is incorrect; it should read “four (22%) of the studies...”.

**Response to Comment #13:** Thank you for your attention to detail. Line 290 has been corrected to “four (22%) of the studies...”.

**Comment 14:** Line 301 – ref [42], one of the excluded studies, is again cited.

**Response to Comment #14:** Thank you for the comment, reference 41 is the Mauricio and Laranjeira which we included in our analysis.

**Comment 15:** Line 303 –re-calculation is needed: four out of 18 studies equates to 22%, not 19%.

**Response to Comment #15:** Thank you for your comment. We have re-calculated the percentages, and it should indeed be 22% and not 19%.

**Comment 16:** Line 335 – “Ten studies found...that organizational antecedents can be attributed to high presenteeism 336 and absenteeism rates among teleworkers [1,17,19,37–38,40,44,48,49]” cites only 9 studies. Re-checking is needed, with addition of the relevant percentage.

**Response to Comment #16:** Thank you for your comment. We have now indicated the correct number of studies as well as the relevant percentages.

**Comment 17:** Lines 340-341 – here the authors report what seems to be quite a significant finding in that it was mentioned by six studies. Thus, while they have written “Due to high job demands, many workers felt compelled to continue working despite illness [1,17,37,40,43,44]”, this could perhaps be strengthened by changing the wording to something like this: “Six (33%) of the studies found that, due to high job demands, workers felt compelled to continue working despite illness [1, 17, 37, 40,43,44]”.

**Response to Comment #17:** Thank you for the comment. This sentence has been revised accordingly.

**Comment 18:** Lines 360-363 – the authors say “Studies have also demonstrated that access to telework can impact incidence of 361 absenteeism [38]...” [reviewer’s emphasis]. As only one study found this it would be preferable to say “One study by Borge et al [38] assessed the relationship between telework access and sickness absenteeism and found that workers who lack the ability to access telework from home showed a higher likelihood of absenteeism”.

**Response to Comment #18:** Thank you for the comment. This sentence has been revised accordingly.

**Comment 19:** Line 417 – the short sentence “Anxiety and worry related to online presenteeism [1]” can probably be removed as this is already covered in lines 410-412.

**Response to Comment #19:** Thank you for the comment. The sentence has been removed to avoid repetition.

**Comment 20:** Line 469 - Lines 487-488 – the authors should re-check the data and report it correctly; saying “We found that 19 studies included a definition of presenteeism” makes no sense, given that there were only 18 studies in the final analysis.

**Response to Comment #20:** Thank you for your comment. We have now indicated that fourteen (78%) of the included studies included a definition of presenteeism.

**Comment 21:** Line 616 – “Lastly, there were only four out of 21 articles that reported a definition of absenteeism” needs to be fixed to reflect the revised data corpus of 18 articles and how many of the final included studies included a definition of absenteeism.

**Response to Comment #21:** Thank you for the comment, we have revised the sentence, so it states 18 articles as opposed to 21 articles.

**Comment 22:** In the Discussion section there are various issues the authors need to consider to remove ambiguity and sharpen their argument.

**Response to Comment #22:** Thank you for the comment. The discussion has been revised to improve the flow and arguments.

**Comment 23:** Lines 537-538 – “Potential gaps within the literature persist regarding absenteeism definitions as there were only four papers that explicitly defined absenteeism, which warrants further investigation” is a little unclear. Were those four papers included in the revised data corpus or could the authors only find four papers in the broader literature?

**Response to Comment #23:** Thank you for your comment. We have specified in the potential gaps that only four of the 18 papers included in our study did not explicitly define absenteeism.

**Comment 24:** Lines 554-555 – in the assertion “Similarities and differences persisted among the antecedents of presenteeism and absenteeism” it is unclear what is meant by “persisted”. Perhaps “were apparent in the review” would be more appropriate?

**Response to Comment #24:** Thank you for the comment. We have revised the sentence accordingly.

**Comment 25:** Lines 577-578 – the wording “...could impact the environment of workers, which can ultimately impact employees’ decision...” makes it sound like there is a difference between “workers” and “employees”. The authors could keep things simple by saying something like “...could impact the work environment, which can ultimately impact employees’ decision...”.

**Response to Comment #25:** Thank you for the comment. We have revised the sentence accordingly.

**Comment 26:** Lines 606-610 – in dealing with the limitations of their study, the authors must tweak their wording to match their final data corpus. Instead of saying “Firstly, the majority of the studies were conducted in High-Income Countries, primarily in Europe and North America” it would be better to say (if correct) “All of the studies were conducted in Europe and North America, regions which are dominated by High-Income Countries”. Likewise, “This geographical concentration suggests a potential bias towards perspectives and experiences from the Global North. Consequently, there is a significant gap in research from the Global South and, in particular, from Lower- and Middle-Income Countries” could be changed to something like “This geographical concentration suggests a potential bias towards perspectives and experiences from the Global North, with a dearth of research from the Global South where Low- and Middle-Income Countries are more prevalent”.

**Response to Comment #26:** Thank you for the suggestion. We have revised the sentences accordingly.

**Comment 27:** In relation to the Conclusion, this remains unusually long and the authors could certainly pare it down more. They could, for example, remove the following wording from lines 668-670: “i.e., job demands impacted an individual's work environment, which lead to individual adaptations of presenteeism and absenteeism behaviours”. Only their most significant, punchy findings should feature in the conclusion – the substantiating information for these having already appeared earlier in the manuscript.

**Response to Comment #27:** Thank you for the comment. The conclusion has been revised and condensed to present the main information.

**Comment 28:** Finally, here are a few basic grammatical, typographic etc errors which leapt out and need correcting – by no means an exhaustive list:

**Response to Comment #28:** Thank you for the comment. We have corrected the entire manuscript involving grammatical or typographic errors.

**Comment 29:** Line 18 in abstract – “defined as” seems to be in a different font

**Response to Comment #29:** Thank you for the comment. We have corrected the font for “defined as”.

**Comment 30:** Line 44 – “15 -69” should read “15-69”

**Response to Comment #30:** Thank you for your attention to detail. We have corrected it to read “15-69”.

**Comment 31:** Line 52 – clumsy wording, “...due to the COVID-19”. Rather revert to “...due to COVID-19”, or switch to “...due to the pandemic”.

**Response to Comment #31:** Thank you for the comment. We have corrected it to “due to the pandemic”.

**Comment 32:** Line 87 “physically absent to work” is clumsy – preferable would be “physically absent from work” (for consistency with line 85). Also, there is a missing full stop after ref [28].

**Response to Comment #32:** Thank you for the comment. We have addressed those points.

**Comment 33:** A line needs to be added before the heading “2.5 Data Synthesis” (line 184).

**Response to Comment #33:** Thank you for the comment. A space has been added before the heading.

**Comment 34:** Table 2 – a blank line should be added between Grigore et al [25] and Hadi et al [41], for consistency and reader-friendliness; without this, at a glance it looks like only 17 studies are included.

**Response to Comment #34:** Thank you for the comment. We have formatted the table as you suggested.

**Comment 35:** Also in Table 2, the authors should also settle on one style for the larger sample sizes – they use the format 4,329 for Borge et al, then 2530 for Okawara et al and 3532 for Takayama et al, and then 12 354 for Ryoo et al. A comma format (4,329) may be most reader-friendly, but all three styles are acceptable as long as there is consistency.

**Response to Comment #35:** Thank you for the comment. We have made changes to the table accordingly.

**Comment 36:** Line 202 – a space is needed after [25].

**Response to Comment #36:** Thank you for your attention to detail. We have added a space after [25].

**Comment 37:** Line 304 – “understands” should read “understand”.

**Response to Comment #37:** Thank you for your attention to detail. It now reads as “understand” rather than “understands”.

**Comment 38:** Lines 315 and 390 – a space is needed after ref [25].

**Response to Comment #38:** Thank you for your attention to detail. We have added a space after [25].

**Comment 39:** Line 392 – “antecedents” should read “antecedent”

**Response to Comment #39:** Thank you for pointing it out. We have addressed the change

**Comment 40:** Line 401 – the short sentence “Anxiety and worry related to online presenteeism [1]” can be removed as it repeats what appears in lines 394-396.

**Response to Comment #40:** Thank you for the comment. We have removed the sentence accordingly as it is repetitive.

**Comment 41:** Line 402 – “include” should read “including”

**Response to Comment #41:** Thank you for pointing it out. We have made a change accordingly
**Comment 42:** The authors should fully act on the previous recommendation to consistently use the same tense when reporting findings from the included studies – some remain reported in past tense and others in present tense, which gets confusing for the reader. See for instance lines 412-413 “Brosi and Gerpott [38] noted...” versus line 417 “Walker et al [48] notes (sic)...”. Also, the paragraph starting with line 419 contains “Yildirim [36] further explores...” and “Adisa et al [1] also focused on...” [reviewer’s emphases].

**Response to Comment #42:** Thank you for your comment. The manuscript has been revised to keep consistency on the tense used.

**Comment 43:** Line 419 – the dash after ‘presenteeism’ needs to be removed; it probably crept in when an unnecessary comma was deleted.

**Response to Comment #43:** Thank you for the comment. The sentence has been revised accordingly.

**Comment 44:** Lines 427-429 – there is confusion regarding citations; “Mauricio and Laranjeira [25,42] and “Grigore [25,42]” that the authors need to correct.

**Response to Comment #44:** Thank you for your comment. We have corrected the references.

**Comment 45:** Line 434 – “Walker et al [50] notes...” should read “Walker et al [50] note...” or “...noted”.

**Response to Comment #45:** Thank you for the comment. This has been revised accordingly.

**Comment 46:** Also in relation to consistency, the authors should do a search on “covid” and replace each instance with either “COVID” or “COVID-19” across the whole manuscript, for consistency. An exception to this would be in the reference list and where studies which use different versions are directly quoted.

**Response to Comment #46:** Thank you for the suggestion. We have revised the manuscript so that “covid” is replaced with “COVID” or “COVID-19”.

**Comment 47:** Line 479 – insert a blank line before the heading “4.1 Definitions for Presenteeism and Absenteeism”.

**Response to Comment #47:** Thank you for the comment. We have inserted a blank line before the heading “4.1 Definitions for Presenteeism and Absenteeism”.

**Comment 48:** Table 3 – fifth line under sub-themes should read “Poor mental health”, not “Poor metal health”. Okawara et al is incorrectly referenced – [42] instead of [44]. The authors should also remove residual underlining which is not needed.

**Response to Comment #48:** Thank you for the comment. We have revised the manuscript to say, “Poor Mental Health” and changed the reference accordingly.

**Comment 49:** Table 4 – a blank line should be added between themes, to be consistent with Table 3 and for greater reader-friendliness.

**Response to Comment #49:** Thank you for the comment. Blank lines have been added between the themes in Table 3 for greater reader-friendliness.

**Comment 50:** Line 594 – “distinguishing” should read “distinguish”.

**Response to Comment #50:** Thank you for pointing it out. We have addressed it accordingly.

**Comment 51:** Line 598 – “...environment in which one works in...” should be “...environment in which one works...”.

**Response to Comment #51:** Thank you for the comment. This sentence has been revised accordingly.

**Comment 52:** Line 677 – “reduce” should read “reducing”.

**Response to Comment #52:** Thank you for the comment. The sentence has been revised.

**Comment 53:** Reference list and supplementary materials – the authors need to re-check these carefully for typographical errors. For instance, in the tables of included and excluded studies some article titles and author names are in capital letters for no clear reason, there is inconsistent formatting – some two-author papers use “and” between the authors’ names whilst others use “&” and some studies have incomplete titles or inscrutable titles like “attendance” or “ROIDE RR.HH”. Wherever possible, rather than using “N/A” for authors it is also preferable (for credibility and trustworthiness) to name SOME sort of author (eg: an organisation) for each and every article sourced. On the Included Studies table, it is unclear why the word “dissertation” is in red for Michael 2021, and also why no date of data collection is shown – surely this should have been apparent on reading the dissertation?

**Response to Comment #53:** Thank you for the feedback. We have made changes to the reference list to improve accuracy.
