# Supplementary material for: Sick leave or work sick? Examining the antecedents and conceptualizations of presenteeism and absenteeism among teleworkers during COVID-19: A scoping review
Source: PLOS Ment Health. 2025 May 13;2(5):e0000300. doi: 10.1371/journal.pmen.0000300 (PMC12798164; doi:10.1371/journal.pmen.0000300)
Supplement: Attachment — Submitted filename: Response to Reviewers (2).docx [file pmen.0000300.s007.docx]

**Reviewer #3 Comments**

*Abstract*

**Comment 1:** Line 16-18: The definitions of presenteeism and absenteeism are briefly introduced but could benefit from succinctly connecting them to telework.

**Response 1: Thank you for your comment. We have revised lines 19-22 of the abstract to succinctly establish the relationship between presenteeism, absenteeism and telework.**

**Comment 2:** Clarify the specific contribution of the study in addressing gaps in literature on telework-related presenteeism and absenteeism.

**Response 2: We have specified the contributions of this study in addressing gaps in literature on telework-related presenteeism and absenteeism in page 2, lines 16-18.**

*Introduction*

**Comment 3**: Page 4, Lines 60-63: Contradictory findings on productivity are mentioned but not resolved. Clarify how this study contributes to resolving these contradictions.

**Response 3: We have mentioned that this paper will tackle the issue of contradictory findings regarding productivity during telework in page 3, lines 63-65.**

**Comment 4:** Page 5, Lines 75-80: Definitions of presenteeism and absenteeism are presented inconsistently across studies. Consider integrating an overarching conceptual framework/terminology early on.

**Response 4: We appreciate your comment and recognize that the definitions of presenteeism and absenteeism vary across the studies. We have integrated the terminology of presenteeism and absenteeism by moving a paragraph to establish it earlier on page 4, lines 73-88.**

**Comment 5:** Find a way to integrate the paragraph starting lines 72-82 with paragraph starting 89-99.

**Response 5: We have integrated the paragraphs that define presenteeism and absenteeism. It can be found on pages 4-5, lines 88-100.**

**Comment 6:** Enhance the discussion on the relevance of the social determinants of health framework to provide a stronger rationale for its use.

**Response 6: We have revised the introduction and discussion section to indicate the rationale of using the Social Determinants of Health model. Please see page 6 lines 115 to 121, and page 34 lines 589 to 593.**

*Methods*

**Comment 7:** Page 8, Lines 165-175: The exclusion of non-English articles and studies from non-Western regions limits the generalizability of findings. Acknowledge this limitation explicitly in the methods section.

**Response 7: We appreciate your comment and have revised the methods section to explicitly acknowledge this limitation. Please see page 8, lines 171-173**

**Comment 8:** The inclusion/exclusion criteria lack detail on how terms like "telework" were operationalized during screening.

**Response 8: We have taken into account your suggestion and have mentioned how we operationalized telework on pages 7-8, lines 159-161.**

**Comment 9:** Why limit the age range to above 18 years when the literature says 15-69-year-olds work in the telehealth sector in Canada? (line 54)

**Response 9: We have provided a rationale for the age range selected for this study. Please see page 8, lines 168-171.**

**Comment 10:** Provide a more detailed justification for the timeframe (2020-2023) and explain how mixed-methods studies were synthesized.

**Response 10: We have acknowledged this and justified the timeframe for 2020-2023 in a more detailed manner in pages 8-9, lines 183-185. We have also explained how the studies were synthesized in page 14, lines 273-274.**

**Comment 11:** Line 136, what is the registration code/number?

**Response 11: Thank you for your comment. We have included the registration DOI for our protocol registration on Open Science Framework. Please see page 7, lines 138 to 139.**

**Comment 12:** Line 164, edit the highlighted word, “a”.

**Response 12: Thank you for your comment. We have edited the highlighted word “a” on page 8, line 165.**

*Results*

**Comment 13:** Page 15, Table 3: Some sub-themes, such as "online presenteeism," are only superficially addressed in the narrative.

**Response 13: Thank you for your suggestion. However, in this scoping review, we looked at the construct presenteeism in general which mostly occurs in a remote setting with teleworkers.**

**Comment 14:** Page 18, Lines 309-319: The relationship between organizational antecedents and individual behaviours requires further elaboration.

**Response 14: We have elaborated on the relationship between organizational antecedents and individual behaviours in the results section. Please see page 21, lines 327-328.**

**Comment 15:** Expand on the nuanced interplay between telework flexibility and presenteeism, as mentioned in Page 18, Lines 319-324.

**Response 15: We have addressed your feedback by including the flexibility and presenteeism that comes with telework in page 21, lines 333-334.**

*Discussion*

**Comment 16:** Page 29, Lines 531-538: The interconnectedness of telework access, presenteeism, and absenteeism is introduced but lacks actionable strategies for balancing these behaviours.

**Response 16: We recognize the importance of this and have included actionable strategies for balancing these behaviours in the discussion section. Please see page 32, lines 557-564.**

**Comment 17:** Page 31, Lines 563-571: The bidirectional relationship between organizational factors and individual outcomes is not sufficiently supported with examples.

**Response 17: We have expanded on the bidirectional relationship between organizational factors and individual outcomes on page 34, lines 604-606.**

**Comment 18:** Highlight the implications of telework for equity, particularly regarding gender and socioeconomic status, to align findings with broader workplace inclusion goals.

**Response 18: We have discussed the implications of** **telework for equity, with a focus on gender and socioeconomic status, in the discussion section. Please see pages 32-33, lines 565-570.**

*Conclusion*

**Comment 19:** Page 33, Lines 629-631: Future research directions are overly general and do not clearly address gaps identified in the review.

**Response 19: We have revised the future research directions to specifically address the limitations outlined in our review. Please see page 37, lines 658 to 667.**

**Comment 20:** Specify concrete areas for future research, such as demographic disparities in presenteeism or cultural variations in telework outcomes.

**Response 20: Thank you for the recommendation. We have specified concrete areas in the future research section such as examining presenteeism and absenteeism in different cultural and geographic contexts, as well as more studies investigating absenteeism behaviours. Please see page 37, lines 658 to 667.**

*General Comment*

**Comment 21:** Definitions: Ensure consistent use of presenteeism and absenteeism definitions throughout the manuscript. For example, reconcile differences between "online presenteeism" and "working while ill."

**Response 21: We have defined presenteeism as loss of productivity due to working while sick on page 38, lines 695-697, and contrasted the difference between online presenteeism and “working while ill” in page 30, lines 512-513.**

**Comment 22:** Citations: Provide in-text citations for specific claims, especially in the discussion of individual antecedents (e.g., guilt, poor mental health).

**Response 22: We have added in-text citations for specific claims we made in the discussion. Please refer to pages 29 to 32, lines 493 to 555.**

**Comment 23:** Tables and Figures: Improve the clarity of Table 2 by adding a column summarizing key findings for each study.

**Response 23: Thank you for the suggestion. We added a column highlighting each of the study’s findings. Please see Table 2 for the changes.**

**Comment 24:** Limitations: Discuss the absence of studies from the Global South more critically and suggest ways to address this imbalance in future reviews.

**Response 24: We have discussed the absence of studies from the Global South more critically and recommended some ways to address this imbalance. Please see page 35, lines 625-631**

**Comment 25:** Policy Implications: Expand on how findings can inform telework policies in diverse organizational contexts, such as small businesses versus multinational corporations.

**Response 25: We have added to our conclusion by including how telework policies can be utilized in smaller businesses versus multinational corporations. Please see page 38, lines 685 to 693.**

**Reviewer #4 Comments**

**Comment 26:** PubMed is commonly used for scoping reviews due to its broader scope, while MEDLINE, often accessed via platforms like Ovid, is typically preferred for systematic reviews due to its curated and rigorous indexed content. Other databases could be explored, such as EBSCO, LitCovid (subset of PubMed)/COVID-19 Research Database, Google Scholar/ILO. Other key search terms/words could be work from home, workplace productivity, employee health, mental health, burnout, stress, flexible work.

**Response 26: We consulted a librarian regarding the use of PubMed versus MEDLINE, and they advised us that MEDLINE (via OVID) captures more articles that may not be found in PubMed. Additionally, we included databases such as SCOPUS (via EBSCO) and Web of Science to ensure a more interdisciplinary and comprehensive search. The use of Google Scholar is a valuable recommendation. However, we excluded grey literature as part of our exclusion criteria, which is why we did not incorporate this database in our search.**

**Comment 27:** Absenteeism and Presenteeism reasons, Outcomes/Findings/Conclusions could be added to the summary table.

**Response 27: Thank you for the suggestion. We included a column outlining the main findings from the studies. Please see Table 2 for the changes.**

**Comment 28:** Line 182-183: The software version is to be stated.

**Response 28: Thank you for your comment. We have specified what version of the software we utilized for our study. Please see page 9, line 192.**

**Comment 29:** Line 228-229: The sentence is unclear and requires revision.

**Response 29: We have revised the sentence to make it clearer on page 11, lines 238-239.**

**Comment 30:** Line 236: The reason is to be provided.

**Response 30: We have provided a reason as to why 145 studies underwent full-text screening. Please see page 12, lines 245-246.**

**Comment 31:** There were two figures 1, one on Page 12, Figure 1, and the other as an Attachment. Page 12 Figure is to be deleted as it is the uncorrected version.

**Response 31: Thank you for bringing this to our attention. Figure 1 on Page 12 has been removed.**

**Comment 32:** At least one decimal point for the percentage figure is to be presented where applicable in the text and table(s).

**Response 32: We have included one decimal point for the percentage figures where applicable.**

**Comment 33:** Line 306: Cap ‘T ’for table 4

**Response 33: We have fixed the error by making the ‘T’ capital on page 20, line 318.**

**Comment 34:** Table 4 COVID-related Stressors (n=1): There were two references cited. Is it n=1 or n=2? [25] missing for Grigore. ‘n=2’ for ‘Poor Mental Health (2)’

**Response 34: We revised the manuscript to indicate n=2 for the Poor Mental Health theme.**

**Comment 35:** The list of references did not conform to the journal format.

**Response 35: Thank you for your comment. We have revised the references to conform to the journal format (Vancouver).**

**Review #5 Comments**

**Comment 36:** Earlier you stated that the work was done to select the studies for November but line 174 states some papers could be published in December. Was this possible?

**Response 36: We have fixed the date as the search end date was supposed to be December 2023.**

**Comment 37:** Line 399- is the end of the sentence missing?

**Response 37: Thank you for bringing that to our attention. We have fixed the sentence on page 25, in line 416.**

**We want to thank all the reviewers for their suggestions. We believe their feedback has improved the quality of the paper.**
